# Loss of TIP60 (KAT5) abolishes H2AZ lysine 7 acetylation and causes p53, INK4A, and ARF-independent cell cycle arrest

**DOI:** 10.1038/s41419-022-05055-6

**Published:** 2022-07-20

**Authors:** Johannes Wichmann, Catherine Pitt, Samantha Eccles, Alexandra L. Garnham, Connie S. N. Li-Wai-Suen, Rose May, Elizabeth Allan, Stephen Wilcox, Marco J. Herold, Gordon K. Smyth, Brendon J. Monahan, Tim Thomas, Anne K. Voss

**Affiliations:** 1 Walter & Eliza Hall Institute of Medical Research, Melbourne, VIC Australia; 2 Department of Medical Biology, University of Melbourne, Parkville, VIC Australia; 3 Cancer Therapeutics CRC, Parkville, VIC Australia; 4 School of Mathematics and Statistics, University of Melbourne, Parkville, VIC Australia

**Keywords:** Epigenetics, Cell growth

## Abstract

Histone acetylation is essential for initiating and maintaining a permissive chromatin conformation and gene transcription. Dysregulation of histone acetylation can contribute to tumorigenesis and metastasis. Using inducible cre-recombinase and CRISPR/Cas9-mediated deletion, we investigated the roles of the histone lysine acetyltransferase TIP60 (KAT5/HTATIP) in human cells, mouse cells, and mouse embryos. We found that loss of TIP60 caused complete cell growth arrest. In the absence of TIP60, chromosomes failed to align in a metaphase plate during mitosis. In some *TIP60* deleted cells, endoreplication occurred instead. In contrast, cell survival was not affected. Remarkably, the cell growth arrest caused by loss of TIP60 was independent of the tumor suppressors p53, INK4A and ARF. TIP60 was found to be essential for the acetylation of H2AZ, specifically at lysine 7. The mRNA levels of 6236 human and 8238 mouse genes, including many metabolism genes, were dependent on TIP60. Among the top 50 differentially expressed genes, over 90% were downregulated in cells lacking TIP60, supporting a role for TIP60 as a key co-activator of transcription. We propose a primary role of TIP60 in H2AZ lysine 7 acetylation and transcriptional activation, and that this fundamental role is essential for cell proliferation. Growth arrest independent of major tumor suppressors suggests TIP60 as a potential anti-cancer drug target.

## Introduction

Chromatin modifications affect DNA accessibility and are essential for the activation of gene expression [1–6]. Histone lysine acetylation mediated by lysine acetyltransferases (KATs) is one of the most abundant histone modifications and is generally associated with actively transcribed gene loci [7–9]. Genetic alterations of genes encoding histone acetyltransferases are found in a wide range of cancers [10]. Targeting the histone acetyltransferases KAT6A and KAT7, members of the MYST family of histone acetyltransferases has emerged as a possible avenue for the treatment of cancer [11, 12].

HIV tat interacting protein, 60 kDa (TIP60) is another member of the MYST family of histone acetyltransferases. TIP60 has been proposed to be involved in a range of cellular processes, including embryonic stem (ES) cell self-renewal [13], DNA damage response [14, 15], transcriptional co-activation, acetylating both histones [16], and transcription factors, including the MYC oncoprotein [17, 18], steroid hormone receptors [19] and the tumor suppressor p53 [20, 21].

TIP60 has been reported to acetylate histone H2A on lysines 5 and 15 (H2AK5, H2AK15) as well as H2Av, H4K5, H4K8, H4K12, H4K16, and H3K14, largely in cell-free and RNAi experiments [16, 22–25]. In contrast, other MYST histone acetyltransferases appear to have a more specific histone acetylation activity in vivo [26–32].

In the context of HIV infection, TIP60 appears to promote cell death, and HIV-1 tat binding to TIP60 facilitates its degradation resulting in impaired TIP60-mediated apoptosis [33, 34]. Overexpression of catalytically inactive TIP60 leads to defects in DNA repair and decreased apoptosis upon γ-irradiation [14]. Similarly, siRNA knockdown of TIP60 impairs the UV-induced DNA damage response and apoptosis [35]. Relevant to the regulation of apoptosis, TIP60 has been reported to acetylate p53 at lysine 120, which is thought to be required for p53-induced apoptosis [20, 21]. In contrast, germline deletion of *Tip60* in mice causes developmental arrest and apoptotic cell death in peri-implantation mouse embryos [36], indicating that in this instance TIP60 is required for cell survival. The conflicting reports on the role of TIP60 in promoting apoptosis or cell survival have not been resolved.

TIP60 appears to either promote or retard cancer progression. Heterozygous loss of *Tip60* accelerates tumor progression in mouse models of breast cancer [37] and lymphoma [38]. Reduced levels of TIP60 are associated with metastatic colorectal cancer [39], metastatic gastric cancer [40], and with poor outcomes in melanoma patients [41]. In contrast, in prostate cancer, high levels of nuclear TIP60 correlate with hormone treatment-refractory disease [42]. The seemingly contradictory reports of TIP60 in various cancers may reflect context-specific functions, affected by parameters such as cancer type and stage, or they may result from changes in TIP60 levels/function that are a consequence rather than a cause of certain oncogenic processes.

Here we used inducible genetic deletion of the *TIP60* gene in human and mouse cells to investigate its role in cell proliferation, cell death, histone acetylation, and transcriptional regulation.

## Materials and methods

### Mice

*Tip60*^*fl/+*^ mice were generated by CRISPR/Cas9 on a *C57BL/6 J* background, using techniques previously described [43, 44]. Briefly, two sgRNAs of the sequence GTACGGAGATGATCCGGGCG and TGGAAGCTACGCCTGCAACT were used to insert *loxP* sites flanking exons 3 and 4 of the *Tip60* gene (Figure S1). The deletion of exon 3 and 4 is predicted to result in a frameshift and disruption of proper TIP60 protein synthesis. Successful targeting was confirmed by sequencing. *Tip60*^fl/+^ mice were crossed to *Rosa26-CreERT2* transgenic mice [45] on a *C57BL/6* background and used to isolate *Tip60*^*fl/fl*^*;ERT2* MEFs.

*Trp53*^*–/–*^ mice [46] and *Cdkn2a*^*–/–*^ mice [47] were used to isolate MEFs. All mice were on a *C57BL/6* inbred background.

Male and female young adult mice were used. Mice were genotyped by PCR; details are described in the supplement. Genotyping primers are listed in the supplement Methods Table 1.

Experiments were performed with the approval of the Walter and Eliza Hall Institute for Medical Research Animal Ethics Committee and according to the Australian code of practice for the care and use of animals for scientific purposes.

### Cells

Murine embryonic fibroblasts (MEFs) were cultured in MEF medium (DMEM, 10% FBS, and 2 mM GlutaMAX^TM^) at 37 °C, in 5% CO_2_, and 3% O_2_ (near-physiological tissue oxygen concentration). HEK293T, HEK293, and U2OS cells were cultured in MEF medium at 37 °C and 5% CO_2_. To induce recombination of the *Tip60*^fl^ locus, *Tip60*^*fl/fl*^*;ERT2* and *Tip60*^*+/+*^*;ERT2* control MEFs were treated with 250 nM 4-OHT for a maximum of 5 days, if not indicated otherwise, and consequently grown in 4-OHT free MEF medium.

### MEF isolation

Briefly, E13.5 embryos were dissected and washed in PBS. Head, extremities, and liver were removed, and the torso was collected in MEF medium, supplemented with 1% (v/v) penicillin and streptomycin (Gibco, 15140), and minced by pipetting several times consecutively through 18 G, 21 G, and 24 G needles. The cells were centrifuged with low speed (500 × *g*) for 5 min and resuspended in warm MEF medium supplemented with penicillin and streptomycin and plated onto gelatin-coated 10 cm Petri dishes.

### Inducible *Tip60* CRISPR knockout cell lines

Dox-inducible CRISPR knockout cell lines were generated as described [48, 49]. Two single guide RNAs, sgRNA1 GCCGGCACCGCCTCAAGCCG and sgRNA2 CTGCCTCCCTACCAGCGCCG, were used to target exons 8 and 11 of the human *TIP60* gene and murine *Tip60* gene. Cells were treated with 1 μg/ml dox for the indicated times. CRISPR indels were analyzed using the primers displayed in the supplement Methods Table 2. Indel frequencies were determined by NGS and calculated as a proportion of sequences with indels of all sequences obtained. Only sequences with at least 25 reads were included in this calculation.

### SDS-PAGE and immunoblotting

Detailed protocols for acid histone extraction and quantification, cell fractionation protein extraction, and SDS-PAGE and immunoblotting can be found in the supplement. Histones were acid extracted, as described [50] with slight modifications. Cells were fractionated using a subcellular protein fractionation kit for cultured cells (Thermo Scientific, 78840) according to the manufacturer’s instructions. SDS-PAGE and immunoblotting were performed using the Li-Cor system (LI-COR Biosciences), details are described in the supplement. Antibodies are listed in the supplement Methods Table 3.

### SA-β-galactosidase staining by X-gal

SA-β-galactosidase staining was performed with a senescence β-galactosidase staining kit (CST, 9860) according to the manufacturer’s instructions. SA-β-galactosidase positive and negative cells were counted in triplicate in three images with at least 300 nuclei per replicate using ImageJ. Cells were irradiated with 32 Gy and cultured for 5 days prior to staining as positive controls.

### RT-qPCR

RT-qPCRs were performed in triplicate. RNA was isolated using RNeasy Kit (Qiagen, 74106) according to the manufacturer’s instructions. Details of the RT-qPCR procedure can be found in the supplement. RT-qPCR primers are listed in the supplement Methods Table 4.

### RNA-seq

RNA-seq was performed on *Tip60*^*fl/fl*^*;ERT2* and *Tip60*^*+/+*^*;ERT2* MEFs induced with 4-OHT for 3 days and 5 days, as well as HEK293g1/C9, HEK293g2/C9, and guide-only HEK293g1 and HEK293g2 controls induced with dox for 3 days. RNA-seq was performed on 2–3 replicates each of U2OSg1/C9, U2OSg2/C9, and guide-only U2OSg1 and U2OSg2 control cells, in total 6 replicates of *TIP60* deleted cells and 5 replicates of control cells, induced with dox for 4 days and with *D. melanogaster* S2 cell spike-in. Details of the RNA-seq procedure can be found in the supplement.

### CUT&Tag sequencing

CUT&Tag was performed on replicate cultures and 100,000 cells per sample, 43,000 human cells each of U2OSg1/C9 and U2OSg2/C9 either treated with doxycycline for 4 days (KO) or untreated (CTL) and 57,000 *D. melanogaster* cells as a spike-in, in total two replicates of *TIP60* deleted cells and two replicates of control cells, as described by the Henikoff laboratory [51] with slight modifications and described in detail in the supplement. The CUT&Tag method produces results similar to ChIP-seq. Briefly, cells are bound by magnetic beads, permeabilized, and subjected with intervening wash steps to incubation with a primary antibody against the target of interest (e.g., H2AZ), followed by a secondary antibody incubation and then pAG-conjugated transposase enzyme (pAG-Tn5). pAG-Tn5 binds to the antibody (target) and is activated. Tn5 cuts the DNA and inserts DNA oligomers (previously loaded on the Tn5) site-specifically into the genome and thereby produces genomic fragments near the position of the target protein. Oligomer-tagged (“tagmented) fragments are recovered, amplified, and sequenced. *D. melanogaster* S2 cells as a spike-in was used to enable normalization and quantification of potential global changes in read coverage between control and *TIP60* deleted cells.

### Flow cytometry analysis

For flow cytometry analysis, including BrdU incorporation, live/cell death cell assay, and γH2AX staining, cells were collected, stained, and analyzed as described in the supplement.

### Live-cell imaging

Cells were imaged using a live-cell imaging system (IncuCyte S3) at 37 °C and 5% CO_2_ with a CMOS camera and ×10 objective for up to 4 days. For 3D confocal live-cell time-lapse imaging, cells were grown in DKSH Ibidi 8-well chamber (DKSH, 80826) and stained with 1 μM SiR-DNA stain and 1 μM verapamil (Spirochem, SC-007). Cells were imaged every 5 min for a total of 24 h using a Leica SP8 and images were analyzed using ImageJ.

### Statistical analysis

Statistical analysis was carried out using the Prism v8.0a software. Statistical methods are stated in the figure legends. Data are presented as mean ± SEM, unless indicated otherwise.

## Results

To assess the role of TIP60 we selected cell types to capture human and mouse cells, normal cells (mouse embryonic fibroblasts, MEFs), immortalized cells (HEK293), and cancer cells (U2OS). U2OS cells are osteosarcoma cells, that can be considered as a cancerous equivalent of primary isolates of fibroblasts [52]. Like MEFs, HEK293 and U2OS cells are of mesodermal origin.

### *Tip60* deletion in mouse fibroblasts causes cell cycle arrest

**Fig. 1:**
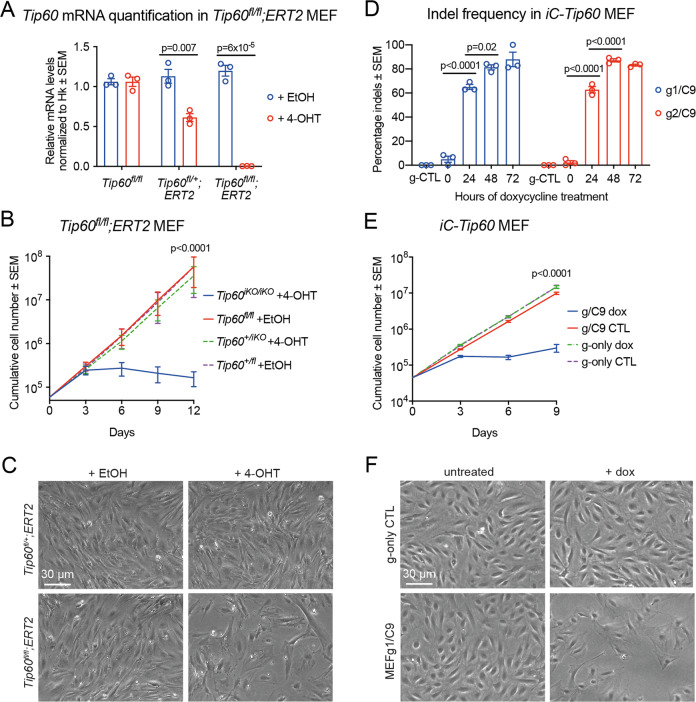
*Tip60* deletion in mouse fibroblasts causes cell cycle arrest. **A** RT-qPCR assessment of *Tip60* mRNA levels normalized to the housekeeping (Hk) gene *Hsp90ab1* in *Tip60*^*fl/fl*^*;ERT2* MEFs induced with 4-hydroxy-tamoxifen (4-OHT) for 3 days, or treated with ethanol (EtOH) vehicle control (means ± SEM, *n* = 3 cell isolates from individual embryos per genotype, unpaired two-tailed *t* test). **B** Proliferation of *Tip60*^fl/fl^*;ERT2* MEFs. Knockout of *Tip60* was induced (*Tip60*^*iKO*^) by treatment for 3 days with 4-OHT, or cells were treated with EtOH vehicle control before culture in normal growth medium (means ± SEM, *n* = 3, unpaired two-tailed *t* test on slopes of log-transformed values). **C** Representative phase-contrast images of *Tip60*^*fl/fl*^*;ERT2* MEFs 5 days after 4-OHT induction vs. heterozygous and EtOH controls. **D** Quantification of indel frequencies after doxycycline (dox) induction of sgRNA expression in *iC-Tip60* MEFs for sgRNA#1 (g1/C9) and sgRNA#2 (g2/C9) and sgRNA-only controls (g-only CTL) (means ± SEM, *n* = 3, one-way ANOVA). **E** Proliferation of *iC-Tip60* MEFs induced to deleted *Tip60* with dox treatment vs. untreated cells (expressing Cas9 only) and g-only CTL controls (means ± SEM, *n* = 3, unpaired two-tailed *t* test on slopes of log-transformed values). **F** Representative phase-contrast images of *iC-Tip60* MEFs 5 days after dox induction vs. untreated cells (expressing Cas9 only) and g-only CTL controls. Circles represent individual data points of cell isolates from individual embryos (**A**, **D**).

Conditional *Tip60* knockout mice were generated (Figure S1A) and crossed to *Rosa26-CreERT2* (*ERT2*) mice [45]. *Tip60*^*fl/fl*^*;ERT2* mouse embryonic fibroblasts (MEFs), *Tip60*^*fl/+*^*;ERT2* MEFs, and *ERT2* control MEFs were isolated and induced with 4-hydroxy-tamoxifen (4-OHT) for 1–5 days resulting in deletion of exon 3 and 4 and a frameshift of *Tip60* (*Tip60*^*iKO/iKO*^*;ERT2* Figure S1B). No *Tip60* mRNA could be detected in *Tip60*^*iKO/iKO*^*;ERT2* MEFs after 3 days of 4-OHT treatment (Fig. 1A).

Treatment of *Tip60*^*fl/fl*^*;ERT2* and *Tip60*^*fl/+*^*;ERT2* MEFs with 4-OHT for 3 days followed by passaging in culture for 2 weeks in the absence of 4-OHT resulted in complete growth arrest in *Tip60*^*iKO/iKO*^*;ERT2* MEFs, whereas heterozygous *Tip60*^*+/iKO*^*;ERT2* control MEFs continued to proliferate normally (Fig. 1B). *Tip60*^*iKO/iKO*^*;ERT2* MEFs retained the ability to adhere to the cell culture plastic after passaging and no excessive floating cells were observed, suggesting an absence of cell death. *Tip60*^*iKO/iKO*^*;ERT2* MEFs displayed a large and flat morphology typical of senescent MEFs (Fig. 1C; compare to [53, 54].

In parallel, we deleted *Tip60* by inducible CRISPR/Cas9 genome editing, involving doxycycline (dox) inducible sgRNA expression marked with GFP, constitutively expressed mCherry-tagged Cas9 [48], using two sgRNAs targeting exons 8 and 11 of *TIP60* in human and mouse cells (Figure S1C, D; referred to as g1/C9 and g2/C9 or g/C9 for merged data). Cell lines containing only sgRNA, lacking Cas9 (referred to as g1 and g2 or g-only for merged data) and either treated with dox or not treated (g-only dox or g-only CTL), and cell lines containing the sgRNA and Cas9 constructs, but not induced to express the sgRNA (referred to as g/C9 CTL), were used as controls. After dox treatment, insertion and deletion (indels) mutations of *Tip60* were analyzed in the bulk population via high-throughput sequencing (Figure S1E). After 48 h of doxycycline induction, the indel frequency for both sgRNAs plateaued at around 85% in MEFs (Fig. 1D). sgRNA-only controls showed no indel activity. Assessment of the top four predicted off-target loci for each sgRNA, identified by WTSI Genome Editing (WGE) [55], showed no indel activity (Figure S1F). RT-qPCR analysis showed a reduction of *Tip60* mRNA in bulk MEFgC/9 cultures after 3 days of induction (Figure S1G); however, small indels are expected to cause a loss of function even without a reduction in mRNA. Currently commercially available TIP60 antibodies (Invitrogen (PA5–23290), Sigma (SAB4500118), Abcam (ab135490), SantaCruz (sc-166323), CST (12058 S)) did not detect endogenous TIP60 protein in our experiments, preventing us from assessing TIP60 protein levels.

The inducible CRISPR *Tip60* mutated MEFs (*iC-Tip60*) were continuously cultured in dox supplemented medium for 9 days. *iC-Tip60* cells displayed growth arrest after 3 days of dox treatment compared to controls (Fig. 1E). After 5 days of doxycycline treatment, *iC-Tip60* MEF morphology closely resembled ERT2-mediated *Tip60* knockout MEFs (Fig. 1F).

The slight outgrowth of cells observed after 9 days of dox treatment was consistent with an approximately 85% efficiency of the CRISPR/Cas9 genome editing system (Fig. 1D). Analysis of cells grown for an extended period in dox supplemented medium revealed a partial loss of sgRNA or Cas9 expression (Figure S2). Cells that either did not integrate or lost or silenced the sgRNA or Cas9 constructs would be expected to grow similarly fast as controls.

Although TIP60 has been reported to play a central role in the DNA damage response [14, 15], we did not observe an increase in γH2AX staining, used as an indication of DNA damage, in *Tip60*^*iKO/iKO*^*;ERT2* MEFs 3 days after 4-OHT induction (Figure S1H).

### *TIP60* deletion in human cells causes cell cycle arrest and failure to progress to metaphase

**Fig. 2:**
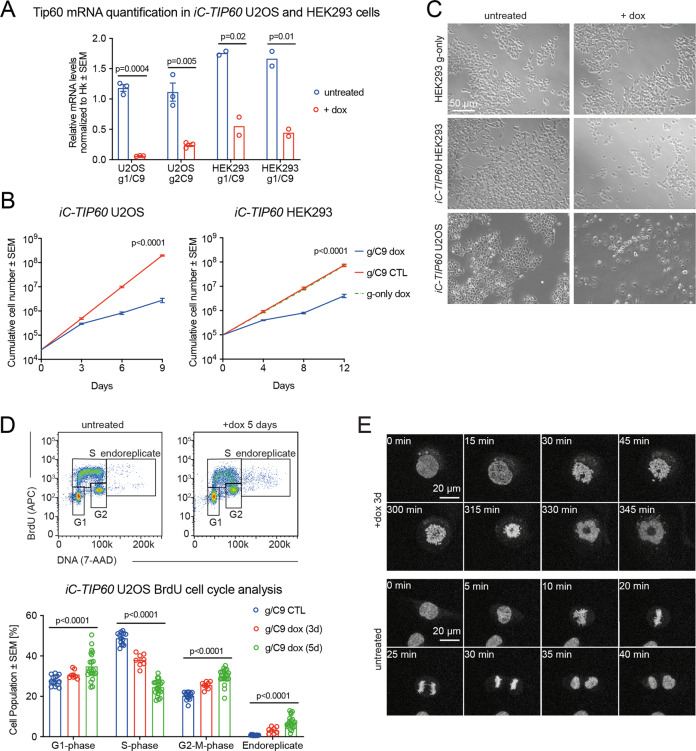
*TIP60* deletion in human cells causes cell cycle arrest with a failure to progress to metaphase. **A** RT-qPCR assessment of *TIP60* mRNA levels normalized to the Hk gene *Hsp90ab1* in *iC-TIP60* U2OS and HEK293 cells induced with dox for 3 days vs. untreated control cells expressing Cas9 only (means ± SEM; *n* = 2, unpaired two-tailed *t* test). **B** Proliferation of iC-*TIP60* U2OS and HEK293 cells vs. untreated control (g/C9 CTL, Cas9 expressing only) and g-only CTL controls (means ± SEM, *n* = 4, unpaired two-tailed *t* test on slopes of log transformed values). **C** Representative phase-contrast images of *iC-TIP60* U2OS and HEK293 cells 5 days after dox induction vs. untreated control (Cas9 expressing only) and g-only CTL control. **D** Flow cytometric cell cycle analysis of BrdU incorporation and DNA content of *iC-TIP60* U2OS cells vs. untreated control cells (g/C9 CTL, Cas9 expressing only). Cells with >4n DNA content suggested endoreplication cells. (Means ± SEM, *n* = 4, two-way ANOVA). **E** Representative maximum intensity projection still images of 3D confocal live-cell time-lapse imaging of *iC*-*TIP60* U2OS cells after 3 days of dox treatment vs. untreated control (Cas9 expressing only). Further IncuCyte live-cell time-lapse imaging is displayed in Figure S3 and Movies 1A−C. Confocal live-cell imaging is displayed in Movies 2A, B. Assessment of cell death is displayed in Figure S5. Circles represent individual data points of replicate experiments using two sgRNAs (**A**, **D**).

*TIP60* was mutated in U2OS and HEK293 cells using dox-inducible CRISPR/Cas9 genome editing. Continuously culturing the cells in dox resulted in a strong reduction in *TIP60* mRNA after 3 days (Fig. 2A) and a reduction in cell growth (Fig. 2B).

Live-cell imaging and tracking of individual cell divisions on *iC-TIP60* U2OS and control cells revealed that most dox-treated *iC-TIP60* U2OS cells underwent a final cell division before complete proliferation arrest (Figure S3A and Movies 1A–C). Some cells maintained normal division rates, consistent with <100% efficiency of CRISPR/Cas9 genome editing (Fig. 1D, S2A).

Brightfield microscopy of *iC-TIP60* U2OS cells revealed a senescent cell morphology. In contrast, *iC-TIP60* HEK293 cells displayed reduced adherence to the tissue culture surface and largely lost contact after 4–5 days of dox treatment (Fig. 2C). *TIP60* deletion led to a 2.4-fold (*TIP60*^*iKO/iKO*^*;ERT2* MEFs) to fivefold (*iC-TIP60* U2OS cells) increase in senescence-associated-β-galactosidase (β-gal) positive cells (Figure S3B). Further analysis of senescence markers revealed only a partial senescence footprint, with increased levels of p21 and p53 in the nucleus of *iC-TIP60* U2OS cells, while other previously reported senescence-associated markers, such as TNF-α, lamin B1, MMP-3, RB, γ-H2AX, and macroH2A remained unchanged and IL-6 was not expressed in U2OS cells (Figure S4A–D). *CDKN2A* (coding for p16 and p19) is deleted in U2OS cells [56] and therefore could not be used as a senescence marker in these cells.

Three different tests assessing cell death (live/dead cell assay, TMRE, and annexin V flow cytometric analysis), showed that cell survival was not affected in U2OS or adherent and floating HEK293 cells after 3 days of doxycycline treatment (Figure S5A–C).

Loss of TIP60 in *iC-TIP60* U2OS cells led to a twofold decrease in S-phase, a 47% increase in G2/M-phase cells, a 34% increase in G1 phase cells, and an eightfold increase in cells with a DNA content >2 N, indicating endoreplication as assessed by DNA content and BrdU incorporation (Fig. 2D). High-resolution 3D confocal live-cell imaging of *iC-TIP60* U2OS cells revealed severely disturbed mitoses with chromosome condensation prolonged from the 25 min (control) to 4 h 45 min during mitosis, failure to arrange chromosomes in a metaphase plate and ultimate failure to divide (Fig. 2E and Movies 2A, B).

### TIP60 is essential for histone H2AZ lysine 7 acetylation

**Fig. 3:**
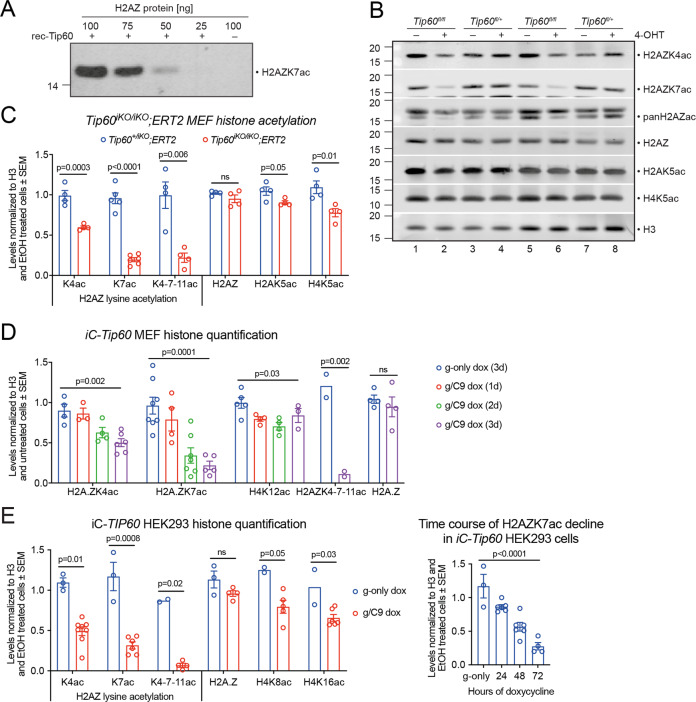
TIP60 is essential for histone H2AZK7 acetylation in human and mouse cells. **A** Cell-free acetylation assay analyzed by Western blot shows full-length recombinant H2AZ histone, with (lane 1–4) and without (lane 5) recombinant TIP60 (rec-Tip60). **B** Representative Western blots of acid extracted proteins from *Tip60*^*fl/fl*^*;ERT2* MEFs treated with vehicle (EtOH, −) or 4-OHT (+) for 3 days and probed for histone and histone acetylation levels as indicated. Total H3 was used as a loading control. **C** Fold change of histone lysine acetylation levels normalized to total H3 protein after 3 days of EtOH or 4-OHT treatment. Shown are *Tip60*^*+/iKO*^*;ERT2* control MEFs and *Tip60*^*iKO/iKO*^*;ERT2* MEFs. **D** Fold change of histone lysine acetylation levels normalized to H3 between *iC-Tip60* and control MEFs. **E** Fold change of histone lysine acetylation levels normalized to H3 between *iC-TIP60* and control HEK293 cells after 3 days of dox treatment and time-course of H2AZK7ac decline. Data from sgRNA#1 and sgRNA#2 were combined. Means ± SEM of 2 to 8 independent experiments are shown (**C**–**E**). Circles represent individual data points of replicates (**C**–**E**). Data were log-transformed and analyzed by unpaired two-tailed *t* test (**C**, **E** left panel) or one-way ANOVA with Benjamini and Hochberg correction (**D**, **E** right panel). Experiments assessing acetylation of histones H2A, H3, and H4 at specific lysine residues are displayed in Figure S6 and full-size Western blots are displayed in the supplemental information.

We assessed histone acetylation at lysines that had previously been proposed to be the histone acetylation targets of TIP60, including H2A-K5, H2A-K15, H4K5, H4K8, H4K12, H4K16 and H3K14 [16, 22–25], as well as lysines on H2AZ, a homolog of *Drosophila melanogaster* H2Av and *S. cerevisiae* Htz1 [57, 58], which were reported to be TIP60 acetylation targets [24, 59, 60]. Cell-free acetylation assays with recombinant human TIP60 and histone H2AZ confirmed that the anti- H2AZK7ac antibody used recognized acetylated H2AZ and did not cross-react with unmodified H2AZ (Fig. 3A). Western blotting of acid extracted proteins from *Tip60*^*iKO/iKO*^*;ERT2* and control MEFs 3 days post 4-OHT induction (Fig. 3B) showed that loss of TIP60 caused a 5-fold reduction in pan-H2AZK4-7-11ac, 5-fold in H2AZK7ac, and 2-fold reduction in H2AZK4ac levels (Fig. 3C). Total H2AZ protein levels remained unchanged. Other proposed TIP60 histone acetylation targets showed no changes, apart from a 20% reduction in H4K5ac (Fig. 3C, S6A). No differences were observed between heterozygous *Tip60*^*+/iKO*^*;ERT2* MEFs and wild-type controls.

iC-*Tip60* MEFs showed a progressive decrease in H2AZK7ac over 3 days to sevenfold and a twofold decline in H2AZK4ac (Fig. 3D). The rapid onset of reduction in H2AZ acetylation after genomic deletion of *Tip60* supports a direct dependence on TIP60. We further observed a ~29% reduction in H4K12ac, but no other changes (Fig. 3D, S6B).

Similarly, *TIP60* mutation in HEK293 cells caused a 4-fold reduction in H2AZK7ac, a 2-fold decrease in H2AZK4ac, and a 13-fold reduction H2AZK4-7-11ac (Fig. 3E). H4K16ac was reduced by 37% (Fig. 3E), but other histone lysine acetylation levels were unchanged (Figure S6C).

**Fig. 4:**
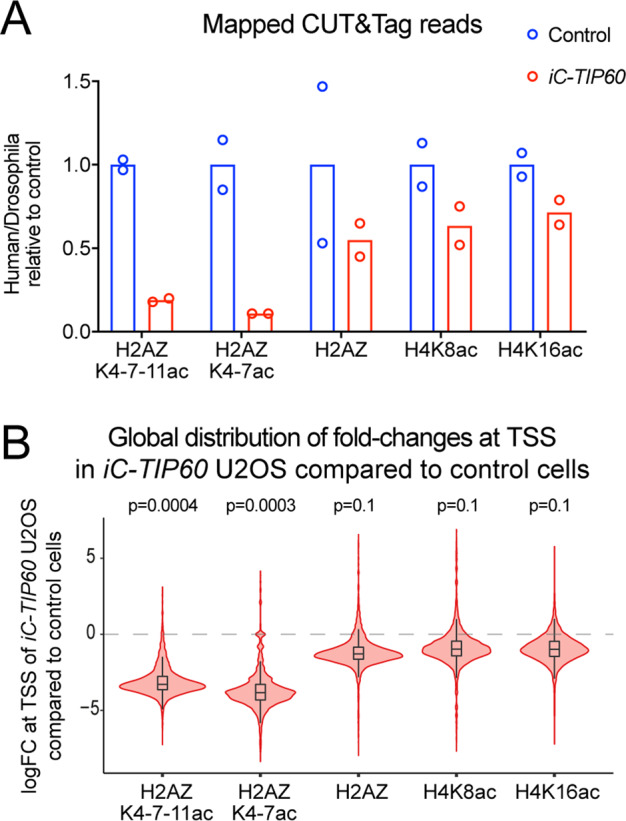
TIP60 is essential for histone H2AZ acetylation at the TSS. **A** Total genome-wide CUT&Tag sequencing reads in *iC-TIP60* U2OS and control cells as a fraction of spike-in S2 *D. melanogaster* cells normalized to control U2OS cells using antibodies detecting H2AZK4-7-11ac, H2AZK4–7ac, total H2AZ, H4K8ac, and H4K16ac (means, *n* = 2, circles represent individual data points of replicates). **B** CUT&Tag sequencing reads quantified in a window ±1000 bp of the TSS of all genes. The distributions of log2 fold changes in read counts (logFC) between *iC-TIP60* U2OS and control cells are displayed as violin plots, with respective box plots inside. Boxes show median and quartiles with boxplot whiskers extending 1.5 inter-quartile ranges beyond the quartiles. *P* values of fry gene set tests for global shifts in occupancy (two-sided). Related data are displayed in Figure S7.

Analysis of H2AZ, H2AZK4–7ac, H2AZK4-7-11ac, H4K8ac, and H4K16ac, levels and distribution in the genome of *iC-TIP60* U2OS cells by CUT&Tag sequencing with *D. melanogaster* S2 cell spike-in revealed a global reduction of H2AZ acetylation by 80–90%, using two separate antibodies against H2AZK4–7ac and H2AZK4-7–11ac (Fig. 4A and S7A). H2AZ and acetylated H2AZ were observed to form peaks over the TSS of genes (Figure S7B), consistent with the previously described patterns [61–69]. Differential occupancy analysis at the TSS of genes (±1000 bp) revealed a global reduction with an average reduction of 90% and 92% in H2AZK4-7-11ac and H2AZK4–7ac, respectively (Fig. 4B). Occupancy of unmodified H2AZ, H4K8ac, and H4K16ac at the TSS remained unchanged. The data point distribution of fold changes for the TSS regions of all genes suggested that histone acetylation changes were not locus-specific, but rather occurred at all or most genes that were decorated with the respective mark (Fig. 4B).

Western blotting of histones and modified histones on the cytosolic, soluble nuclear, and chromatin-bound fractions revealed that chromatin-bound total H2AZ levels remained unchanged in *iC-TIP60* U2OS cells 3 days after dox treatment, while H2AZac levels were reduced by 85% (Figure S8A, B).

### TIP60 is required for normal gene expression in mouse and human cells

**Fig. 5:**
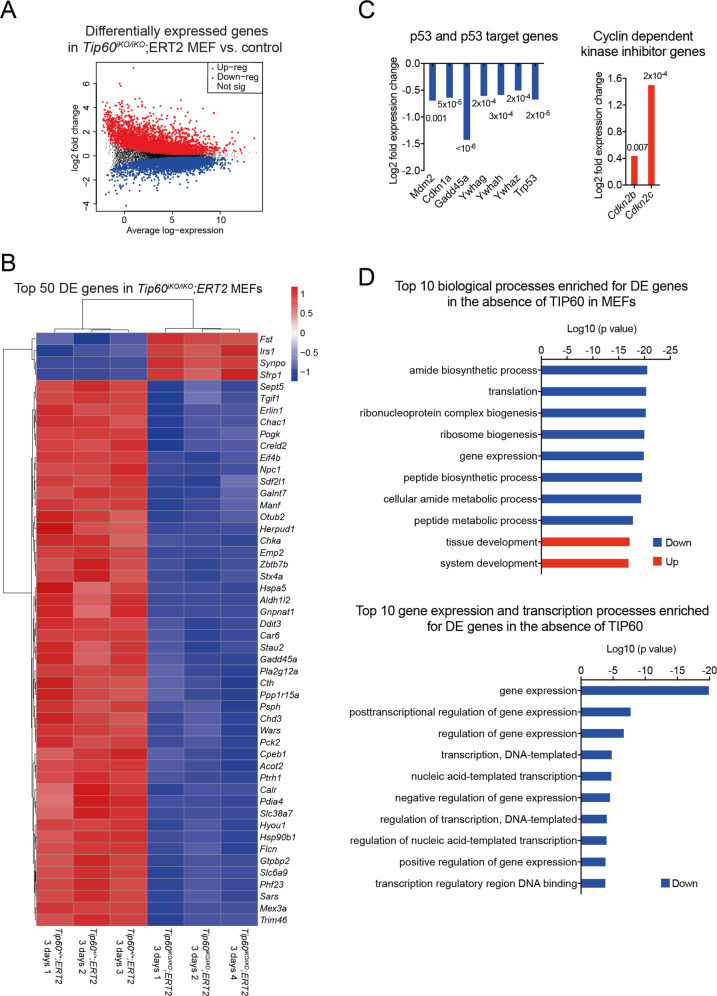
TIP60 is required for normal gene expression in mouse cells. **A**–**D** RNA-seq results of *Tip60*^*iKO/iKO*^*;ERT2* vs. *Tip60*^*+/+*^*;ERT2* control MEFs after 3 days and 5 days (**A**, combined analysis) or after 3 days of 4-OHT treatment **B**–**D** to induced *Tip60* deletion (*n* = 3 cell isolates from individual embryos per genotype). **A** Mean-difference plot showing the log2 fold change vs. average log2 counts-per-million of each mRNA in *Tip60*^*iKO/iKO*^*;ERT2* MEFs vs. *Tip60*^*+/+*^*;ERT2* control MEFs after treatment for 3 and 5 days with 4-OHT. Differentially expressed genes (FDR < 0.05) are indicated. **B** Heatmap of the top 50 differentially expressed (DE) genes by FDR in *Tip60*^*iKO/iKO*^*;ERT2* vs. *Tip60*^*+/+*^*;ERT2* control MEFs after 3 days of 4-OHT treatment. **C** Expression level changes of the *Trp53* (p53) gene and p53 target genes and cyclin-dependent kinase inhibitor genes *Cdkn2b* and *Cdkn2c* in the absence of TIP60. **D** Top 10 biological processes (GO terms) and gene expression and transcription processes (GO terms) enriched for genes differentially expressed (DE) in the absence of TIP60 in MEFs. RNA-seq results of *Tip60*^*iKO/iKO*^ and control MEFs, including genes encoding other histone acetyltransferases, TIP60 complex proteins, cell adhesion proteins, extracellular matrix proteins, and gene expression regulators, are displayed in Figure S9.

Since H2AZ acetylation is reported to play an important role in gene expression in yeast [59], we examined the effect of *TIP60* mutation on transcription. RNA-seq analysis of *Tip60*^*+/+*^*;ERT2* control and *Tip60*^*fl/fl*^*;ERT2* MEFs 3 days and 5 days after 4-OHT treatment revealed 8238 differentially expressed genes in *Tip60*^*iKO/iKO*^*;ERT2* cells compared to control cells (Table S1, Fig. 5A). Differentially expressed genes after 3 days and 5 days were highly correlated (*p* = 5×10^–5^; Figure S9A). For the subsequent analysis, we focused on genes differentially expressed 3 days after induction of *Tip60* deletion to avoid secondary effects of loss of TIP60, at which time a total of 6311 genes were differentially expressed (Table S1). Among the top 50 differentially expressed genes ranked by FDR, 92% were downregulated (Fig. 5B) and among the top 500 differentially expressed genes 77.6% were downregulated, suggesting a bias towards transcriptional repression in the absence of TIP60 (Table S2).

No compensatory upregulation of another *KAT* gene was seen in the absence of TIP60 (Figure S9B). Only minor changes in mRNA levels of the TIP60 protein complex subunits were seen (Figure S9B).

p53 and its target genes, including *Cdkn1a* (encoding p21^CIP1/WAF1^), were generally downregulated in the absence of TIP60 in MEFs (Fig. 5C), suggesting that the p53 pathway was not involved in the cell cycle arrest mechanism caused by the absence of TIP60. Interestingly, *Cdkn2b* and *Cdkn2c* (encoding p15^INK4B^ and p18^INK4C^ respectively) were upregulated (Fig. 5C). Cell adhesion genes and collagen genes were up to 25-fold upregulated (Figure S9C), consistent with a senescent cell phenotype. This notably included cell-substrate adhesion genes, such as integrin and collagen genes, with 30% of the genes within this biological process upregulated (*p* = 0.0002; Table S3).

Among the top 10 enriched biological processes, 8 were downregulated, including gene expression, protein translation, and associated processes (Fig. 5D; Table S3), consistent with cell cycle arrest and reduced requirement for metabolism and protein synthesis after the loss of TIP60. Transcription and gene expression processes overall were downregulated in the absence of TIP60 (Fig. 5D; Table S3). Downregulated pathways included p53-signaling, cell cycle regulation, apoptosis, and necroptosis (Table S4), congruent with the observation that TIP60 knockout MEFs neither proliferate, nor undergo apoptosis or other forms of cell death, but rather enter cell cycle arrest.

In HEK293 cells, the use of sgRNA#1 or sgRNA#2 resulted in highly correlated expression changes 3 days after induction of *TIP60* deletion (Figure S10A) and therefore the data from the two different sgRNAs were combined for all subsequent analyses.

**Fig. 6:**
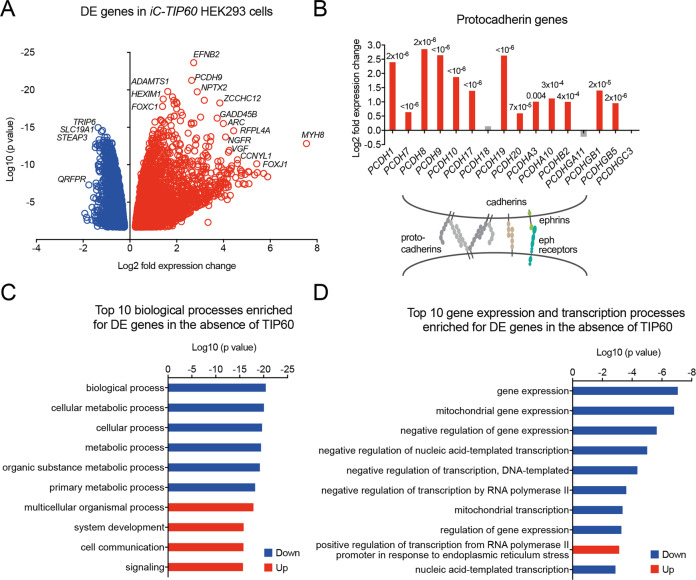
TIP60 is required for normal gene expression in human cells. **A**–**D** RNA-seq results from *iC-TIP60* vs. control HEK293 cells after 3 days of 4-OHT treatment to induce *Tip60* deletion (*n* = 4). **A** Volcano plot displaying genes significantly differentially expressed (DE) in *iC-TIP60* vs. control HEK293 cells (FDR < 0.05). Results from both sgRNAs were similar and were thus combined. **B** Expression level changes of protocadherin genes in *iC-TIP60* vs. control HEK293 and schematic drawing of cell-cell adhesion interactions upregulated in the absence of TIP60. FDR values are indicated in the graph. Gray bars represent genes not significantly changed. **C** Top 10 biological processes (GO terms) enriched among genes differentially expressed (DE) in the absence of TIP60. **D** Top 10 gene expression and transcription processes (GO terms) enriched among genes differentially expressed (DE) in *iC-TIP60* vs. control HEK293 cells. Further RNA-seq data of *iC-TIP60* vs. control HEK293 cells, including genes encoding histone acetyltransferases, TIP60 complex proteins, cell cycle regulators, cell senescence genes, apoptosis regulators, and cell-cell adhesion proteins are displayed in Figure S10.

In the absence of TIP60, 6236 genes were differentially expressed between *iC-TIP60* and control HEK293 cells (Fig. 6A; Table S5).

Similar to MEFs, TIP60 complex subunit and KAT genes were not greatly dysregulated in HEK293 cells lacking TIP60 (Figure S10B). Cell cycle and mitosis processes and genes were predominantly downregulated in the absence of TIP60 (Figure S10C; Table S6). Similar to MEFs, cyclin-dependent kinase genes *CDKN2B and CDKN2C* were upregulated in HEK293 cells lacking TIP60 (Figure S10D).

*TIP60* deleted HEK293 displayed a large proportion of cells that lifted off the cell culture plastic. However, the proportion of dead cells was not increased (Figure S5B), suggesting a primary failure in cell-substrate adhesion. Genes encoding cell-cell adhesion proteins, in particular of the protocadherin, cadherin, and ephrin receptor families were upregulated in the absence of TIP60 (Fig. 6B and S10E, Table S5), suggesting cell-cell adhesion increased as cell-substrate adhesion decreased. Overall, 33% of genes with the annotation “cell-cell adhesion” were upregulated in the absence of TIP60 (Table S6).

Six of the top 10 biological processes were downregulated in HEK293 cells lacking TIP60. These included metabolic processes, whereas organ development processes were upregulated (Fig. 6C, Table S6). Similarly, among the pathways affected, metabolic pathways and the cell cycle were downregulated (Table S7). Gene expression and transcription processes were comprehensively downregulated (Fig. 6D).

We performed RNA-seq on *iC-TIP60* U2OS cells 4 days after induced *TIP60* mutation adding in a *D. melanogaster* spike-in cells to enable detection of global changes in mRNA, as previously described [70]. We found a total of 12716 expressed genes, 2707 genes were downregulated, and 1555 genes were upregulated (Table S8; Figure S11A, S11B), representing a shift towards downregulated genes compared to RNA-seq results from HEK293 cells and MEFs without *D. melanogaster* spike-in normalization, while total mRNA levels remained similar. The directional change and the amplitude of change in gene expression in *iC-TIP60* U2OS cells correlated strongly positively with gene expression changes in *iC-TIP60* HEK293 cells (Figure S11C) and *Tip60*^*iKO/iKO*^ MEFs (Figure S11D).

We integrated the *iC-TIP60* U2OS RNA-seq dataset and CUT&Tag dataset to examine correlations between gene expression and H2AZ occupancy and H2AZ acetylation. H2AZ and H2AZac occupancy was detected in wild-type cells at the start of transcription of more than 99% of expressed genes (Table S9). Of the genes decorated with H2AZ and H2AZac, 35% were differentially expressed in the absence of TIP60, whereas the RNA levels of 65% were not affected by the loss of TIP60. Of the differentially expressed genes, 64% were downregulated in RNA levels (Table S9). In *TIP60* wild-type U2OS cells, RNA levels correlated significantly with H2AZ occupancy (*p* < 10^−6^) and H2AZK4-K7 acetylation levels (*p* < 10^−6^; Figure S12A). Furthermore, downregulation of genes in *iC-TIP60* vs. control U2OS cells correlated significantly with H2AZ (*p* < 10^−6^) occupancy and H2AZK4-K7 acetylation levels in genes that were differentially expressed in *iC-TIP60* vs. control U2OS cells (*p* < 10^−6^; Figure S12B).

### Loss of TIP60 causes cell cycle arrest even in the absence of the cell regulators p53, INK4A, and ARF

**Fig. 7:**
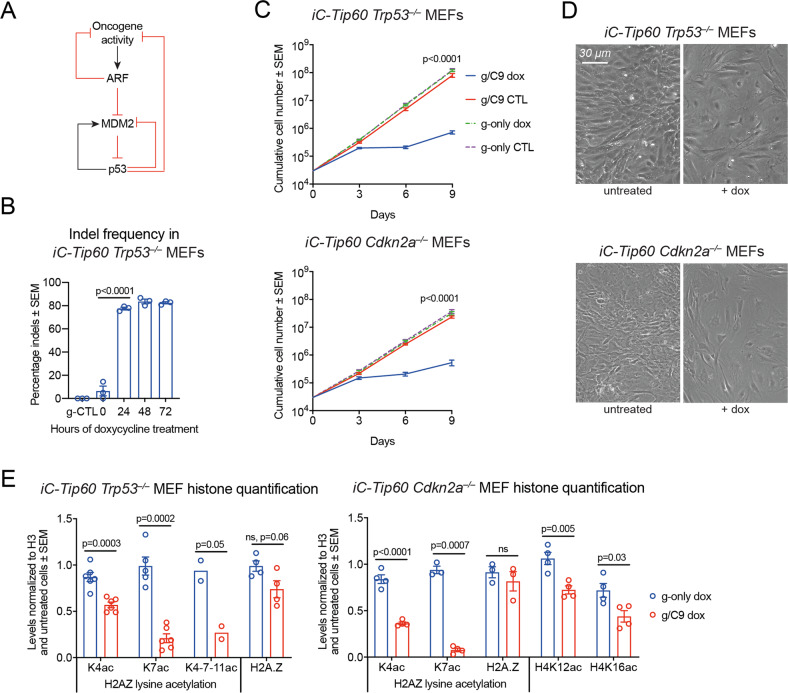
Loss of TIP60 causes cell cycle arrest even in the absence of the cell cycle regulators p53, INK4A and ARF. **A** Simplified schematic displaying the regulatory interplay between ARF, MDM2, and p53. **B** Quantification of indel frequencies in *iC-Tip60 Trp53*^–/–^ MEFs. (Means ± SEM, *n* = 3, one-way ANOVA). **C** Proliferation of *iC-Tip60 Trp53*^–/–^ MEFs and *iC-Tip60 Cdkn2a*^–/–^ MEFs vs. untreated controls (expressing Cas9 only) and g-only controls (means ± SEM, *n* = 6, unpaired two-tailed t test on slopes of log-transformed values). **D** Representative phase-contrast images *iC-Tip60 Trp53*^–/–^ MEFs and *iC-Tip60 Cdkn2a*^–/–^ MEFs 5 days after dox treatment vs. untreated controls. **E** Fold change of total H2AZ and histone acetylation levels normalized to H3 in *iC-Tip60 Trp53*^–/–^ and *iC-Tip60 Cdkn2a*^–/–^ vs. g-only control MEFs after 3 days of dox treatment (means ± SEM, *n* = 2, unpaired two-tailed *t* test on log-transformed values). Experiments assessing histones H2A, H3, and H4 lysine acetylation levels are displayed in Figure S6D.

Our RNA-sequencing results suggested that the ARF-p53 tumor suppressor pathway (Fig. 7A) was not engaged in the cell cycle arrest induced by loss of TIP60 in MEFs. This finding could potentially have bearing on the suitability of TIP60 as a target for cancer therapeutics, as the *TP53* gene is mutated in 42% of cancers [71], and *CDKN2A*, encoding INK4A and ARF, is mutated in 3.6% in a pan-cancer study [71] and in up to 78% in specific types of pediatric leukemia [72]. To formally test if p53 or INK4A-ARF were required for cell cycle arrest induced by loss of TIP60, we deleted the *Tip60* gene in MEFs lacking p53 (*Trp53*^*–/–*^ MEFs) or INK4A-ARF (*Cdkn2a*^*–/–*^ MEFs). In *Trp53*^*–/–*^ MEFg1/C9 cells indel frequency reached 80% after only 24 h of doxycycline induction (Fig. 7B). TIP60 deletion in *Trp53*^–/–^ MEFs or in *Cdkn2a*^–/–^ MEFs resulted in growth arrest, indicating a p53 and INK4A-ARF-independent mechanism of cell cycle arrest (Fig. 7C). The morphology was indistinguishable from *Tip60* only deleted MEFs (Figs. 7D vs. 1C, F).

Similar to wild-type MEFs, deletion of *Tip60* in *Cdnk2a*^*–/–*^ or *Trp53*^*–/–*^ MEFs resulted in a reduction in H2AZK4, H2AZK7, and H2AZK4–11 acetylation (Fig. 7E). In addition to the profound effects on H2AZ acetylation, we found a modest reduction in H4K12ac and H4K16ac in *Tip60* deleted *Cdkn2*^*–/–*^ MEFs compared to control MEFs (Fig. 7E), but not in *Tip60* deleted *Trp53*^*–/–*^ MEFs (Figure S6D).

## Discussion

In this study, we induced *TIP60* deletion in normal mouse cells, immortalized human cells, and human cancer cells, as well as cells lacking the tumor suppressors p53, INK4A, and ARF to investigate the role of TIP60. *TIP60* deletion caused cell growth arrest, but cell survival was not affected.

In our study *loss* of TIP60 caused a p53-independent cell cycle arrest. In contrast, TIP60 has previously been reported to *induce* cell cycle arrest, either as a co-activator of p53 at target genes such as *CDKN1A* (p21^CIP1/WAF1^) [73], or in a p53-independent manner [74] after genotoxic stress in the context of DNA repair. The active role of TIP60 in inducing cell cycle arrest in the context of genotoxic stress appears to involve specific molecular mechanisms, including interaction with DNA damage repair proteins [15]. Our data, showing that the loss of TIP60 causes cell cycle arrest in a range of cell cycle phases, suggest that the observed cell cycle arrest is not due to effects on a single cell cycle regulatory mechanism, but is rather mediated through a broad transcriptional dysregulation of multiple genes required in different phases of the cell cycle for a range of cellular mechanisms, in a manner independent of tumor suppressor and cell cycle regulators. The effects of *TIP60* deletion might differ depending on the phase of the cell cycle when individual cells first experience critically low levels of TIP60 and deficiencies in gene expression. Thus, only a small proportion of cells undergo endoreplication due to the lack of TIP60, while the majority undergo cell cycle arrest without endoreplication. The same effect might be the reason for our observation that not all cells display cellular senescence. γH2AX staining indicative of DNA damage was not increased in *Tip60* deleted MEFs, excluding accumulation of DNA damage as a factor causing cell cycle arrest.

Our data identify the specific lysine residues that rely on TIP60 for acetylation. Loss of TIP60 results in a marked decline in H2AZK7 acetylation and a reduction in H2AZK4 acetylation, consistent with previous reports proposing that TIP60 is required for the acetylation of H2A.Z and gene activation in human and mouse cells [75, 76]. Apart from the profound reduction in H2AZ acetylation, we observed only a modest reduction in acetylation levels at some H4 lysine residues in the absence of TIP60. However, the effects on H4 acetylation were not present in all cell types and the specific H4 residue affected was not consistent between cell types. Various other HATs are known to acetylate H4 lysines [77]. Redundancy between those HATs might conceal a potential effect of loss of TIP60 on global H4 acetylation.

Global chromatin-bound H2AZ levels were not reduced upon *TIP60* deletion in U2OS cells, indicating either no effect of *TIP60* deletion on H2AZ dynamics or a complete halt of H2AZ exchange.

The mitotic defect and increased endoreplication indicate that *Tip60* deleted cells were able to synthesize DNA, but failed to arrange chromosomes in a metaphase plate, and a failure of chromosome segregation. These observations may relate to the knockout phenotype of the yeast homolog of TIP60, *esa1*. *Esa1* null mutant or catalytic mutant *Saccharomyces cerevisiae* display a budding phenotype characterized by incomplete DNA segregation and aberrant spindle orientation [78]. In this context, it is interesting that TIP60-mediated acetylation of aurora B kinase, the catalytic subunit of the chromosome passenger complex, promotes accurate chromosome segregation in mitosis [79].

H2AZ has been shown to play an important role in centromere structure formation [80] and H2AZ depletion has been linked to abnormal nuclear chromatin structure, as well as lagging chromosomes and chromosome bridges during cell division in mammalian cells [81]. The mitosis defect upon loss of TIP60 (and 80% of H2AZac) suggests that TIP60-dependent acetylation of H2AZ may be important in centromere and spindle formation.

Our RNA-seq data on TIP60-deficient cells support a broad role of TIP60 in gene transcription, congruent with the role of H2AZ acetylation. Thousands of genes relied on TIP60 for normal expression levels in MEFs, including genes related to protein translation, ribosome biogenesis, and general gene expression. The *TIP60* mutant cells phenotype is likely to be due to widespread failure of transcription rather than a small subset of genes being responsible for the phenotype. While more genes are downregulated than upregulated in response to the loss of TIP60, the amplitude of change is generally larger in the upregulated genes, suggesting that we also observed gene expression changes secondary to cellular events such as cell cycle arrest. H2AZ acetylation has been correlated with active transcription, consistent with a role of TIP60 in H2AZ acetylation and gene activation, albeit less locus-specific than previously reported [82–84]. H2AZ is found broadly at the transcription start site (TSS) of active and inactive genes as well as in gene bodies of inactive genes [63]. In contrast, acetylated H2AZ is shown to exclusively accumulate at the TSS of active genes [63]. Our data suggest that TIP60 is responsible for the acetylation of H2AZK7 at active genes. A comparison of the RNA-seq and the Cut&Tag data revealed a positive correlation of RNA levels with H2AZ occupancy and H2AZ acetylation. High H2AZ acetylation and H2AZ occupancy correlated with stronger downregulation in gene expression upon *TIP60* mutation. However, RNA levels of 65% of genes displaying decreased H2AZ acetylation at their TSS were not significantly changed in their expression levels after *TIP60* mutation. Residual H2AZ acetylation, i.e., at lysine 4, might be sufficient to keep genes active in the absence of TIP60. Furthermore, high expression levels and thus more open chromatin structure combined with active RNAPII transcription through the locus might facilitate continued transcription even upon loss of H2AZ acetylation. Lastly, H2AZ might only be more important for the onset of gene transcription but not for maintaining open chromatin structure and active gene expression.

In conclusion, we propose a mechanism by which TIP60 acts as a co-activator of gene transcription through H2AZ acetylation. Identifying H2AZK7 and H2AZK4 as endogenous histone acetylation targets of TIP60 in a number of human and mouse cell types is an important step in understanding the role of TIP60 and its histone acetylation function. Furthermore, the finding that loss of TIP60 induces cell cycle arrest independent of the tumor suppressors p53, INK4A, and ARF, and identification of H2AZK7 acetylation as a potential biomarker are essential steps for the assessment of TIP60 as a potential therapeutic target.

## Supplementary information

Supplemental Figures S1-12 and Table S1

Movie 1A

Movie 1B

Movie 1C

Movie 2A

Movie 2B

Dataset S2

Dataset S3

Dataset S4

Dataset S5

Dataset S6

Dataset S7

Dataset S8

Dataset S9

Original Data File

Reproducibility checklist

## Data Availability

Complete results from our RNA-seq studies, including complete lists of differentially expressed genes, differentially enriched GO terms and KEGG pathways, are included in the in the supplementary tables. Raw data NCBI Short Read Archive accession number GSE208383.
