# Supplementary material for: Loss of TIP60 (KAT5) abolishes H2AZ lysine 7 acetylation and causes p53, INK4A, and ARF-independent cell cycle arrest
Source: Cell Death Dis. 2022 Jul 20;13(7):627. doi: 10.1038/s41419-022-05055-6 (PMC9296491; doi:10.1038/s41419-022-05055-6)
Supplement: Supplementary file 1 — Supplemental Figures S1-12 and Table S1 [file 41419_2022_5055_MOESM1_ESM.pdf]

Supplementary Information

Loss of TIP60 (KAT5) abolishes H2AZ lysine 7 acetylation and causes p53, INK4A and ARF-independent cell cycle arrest

Johannes Wichmann[1,2], Catherine Pitt[1,2], Samantha Eccles[1], Alexandra L Garnham[1,2], Connie S.N. Li-Wai-Suen[1,2], Rose May[1], Elizabeth Allan[1,4], Stephen Wilcox[1], Marco J. Herold[1,2], Gordon K. Smyth[1,3], Brendon J. Monahan[1,2,4], Tim Thomas*[1,2,5], Anne K. Voss*[1,2,5]

12 Supplementary Figures (S1 to S12)

9 Supplementary Data Tables (S1 supplied here and S2 to S9 supplied as Excel files)

5 Supplementary Movies (legends supplied here and movies supplied as AVI files)

Supplementary Methods (including 6 Methods Tables supplied here)

Supplementary Figures

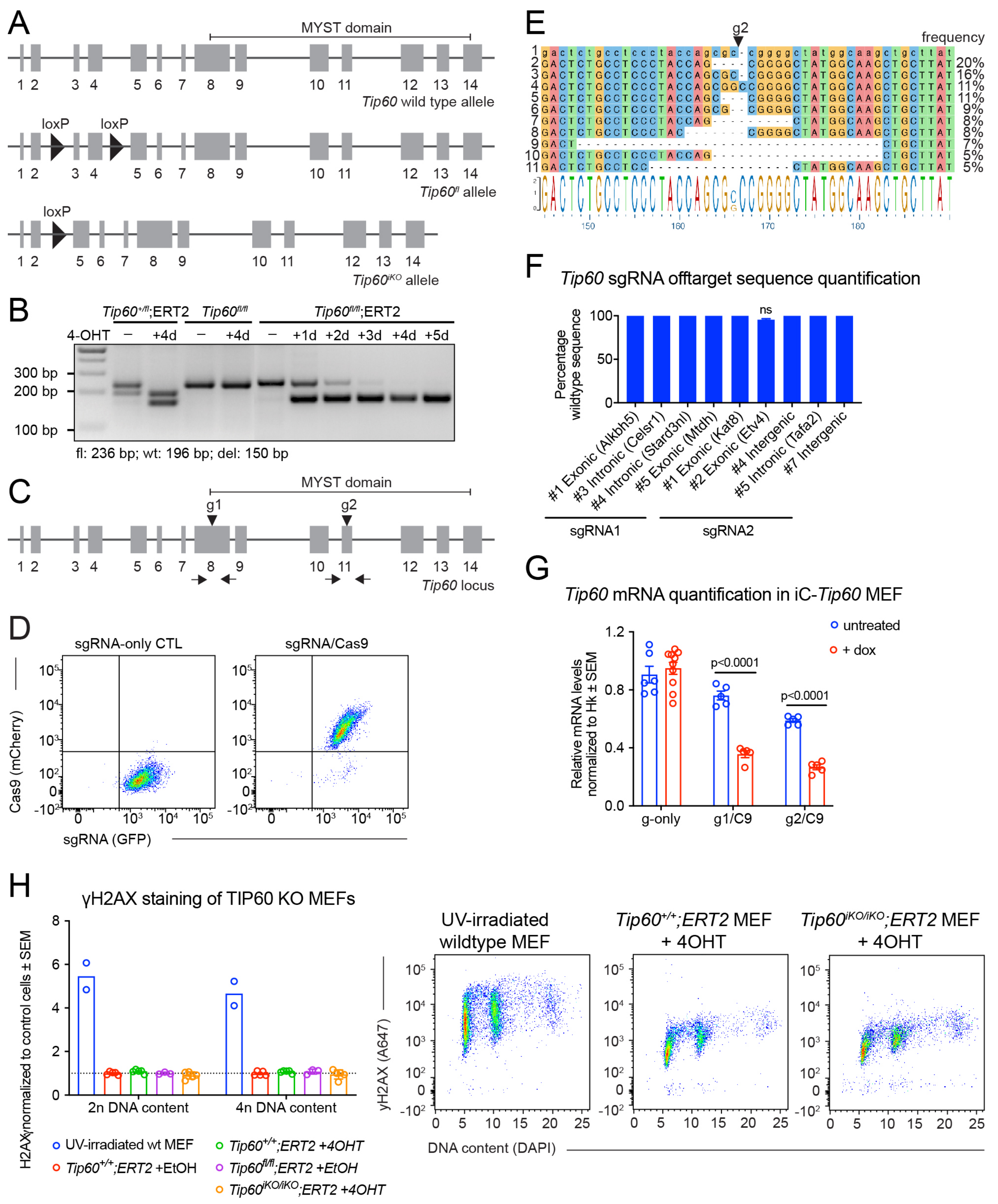

Figure S1. Cre-recombinase and CRISPR/Cas9 meditated, inducible loss of TIP60 in cells does not induce detectable DNA damage.

(A) Schematic drawing of conditional *Tip60* allele. Exons are represented by numbered grey boxes. Exons 3 and 4 are flanked by *loxP* sites (*Tip60^{fl}* allele). In combination with *Rosa26-Cre-ERT2,*

tamoxifen treatment leads to the deletion of exons 3 and 4 of the *Tip60^{fl}* allele resulting in a frameshift and so generating the induced knockout *Tip60^{iKO}* allele.

(B) Representative DNA gel of three-way PCR genotyping of *Tip60^{+}* (wild-type), *Tip60^{fl}*, and *Tip60^{iKO}* (induced knockout) alleles, shown here in 4-OH-tamoxifen (4-OHT) and vehicle (EtOH) treated MEFs. Upon 4-OHT treatment of MEFs, the sequence flanked by *loxP* sites (which yielded a 236 bp band) is excised to produce the deleted allele (yielding a 150 bp band). This process is completed over the course of 3 days.

(C) Inducible CRISPR/Cas9 *Tip60* (*iC-Tip60*) mutation, with sgRNA PAM sites represented by black arrowheads. Upon dox induction, sgRNA#1 or sgRNA#2 is transcribed, leading to CRISPR/Cas9 mediated indels in exon 8 or exon 11, respectively.

(D) FACS of the Cas9 and sgRNA positive MEFs. Cas9 and sgRNAs were co-expressed with the marker proteins mCherry and GFP, respectively, to allow sorting of double positive cells (g/C9) and GFP positive sgRNA-only control cells (g-only CTL).

(E) Indel analysis of *iC-Tip60* MEFs after 3 days of dox induction via high-throughput sequencing. The sequences are displayed in decreasing frequency and aligned to the wild-type sequence (1st row, lower case letters). Indels are shown as a percentage of all sequences with at least 25 reads (total of 502 out of 3052 reads).

(F) Assessment of potential off-target effects. Quantification of wild-type allele frequencies at potential off-target loci for each sgRNA in *iC-Tip60* MEF g1/C9 and g2/C9 after 3 days of dox treatment as a percentage of all sequences with at least 25 reads. Potential off-target sites were identified and ranked by WTSI Genome Editing (WGE) (1). No significant off-target indel activity was detected.

(G) RT-qPCR assessment of *Tip60* mRNA levels, normalized to housekeeping (Hk) gene *Hsp90ab1*, in *iC-Tip60* and control MEFs (g/C9 and g-only control) induced with dox for 3 days. Means ± SEM of n = 3 MEF isolates from individual embryos per genotype, each assessed in triplicate and analyzed by unpaired two-tailed t test.

(H) Quantification of γH2AX as an indicator of DNA damage in *Tip60$^{+/+}$;ERT2* and *Tip60$^{iKO/iKO}$;ERT2* MEFs 3 days after 4-OHT induction or vehicle (EtOH) treatment. Mean fluorescence intensity (MFI) of γH2AX was normalized to EtOH treated *Tip60$^{+/+}$;ERT2* MEFs. UV-irradiated MEFs were used as γH2AX positive controls. Due to increased DNA content of G2/M-phase cells, γH2AX MFI was evaluated separately for 2n and >2n cell population as indicated by DNA stain. No increase in γH2AX was observed in *Tip60* deleted MEFs. Exemplary FACS plots are shown. A minimum of 3 replicates for each genotype were assessed, except for the positive control (n=2).

Circles represent individual datapoints of replicates (G,H).

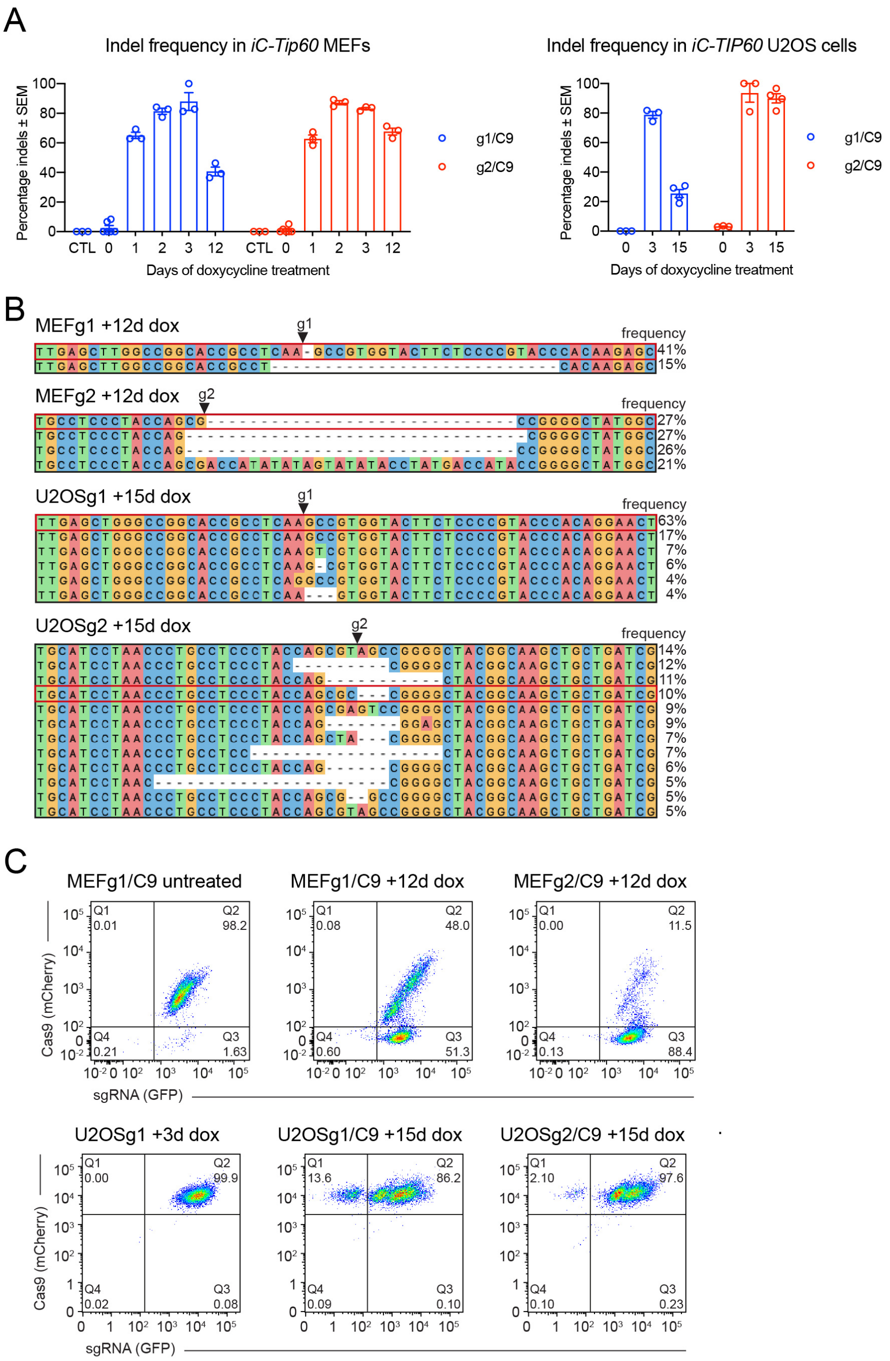

Figure S2. CRISPR/Cas9 indels in *iC-TIP60* cells that return to normal growth rates.

(A) Indel frequencies for *iC-Tip60* MEFs and *iC-TIP60* U2OS cells, including cells cultured in dox supplemented medium for 12 and 15 days, respectively.

(B) Representative indel sequence alignment and frequencies for each unique sequence. The wild-type allele is marked with a red box.

(C) Flow cytometry analysis of GFP and mCherry populations in *iC-Tip60* MEFs and *iC-TIP60* U2OS cells after culture in dox supplemented medium for the duration indicated.

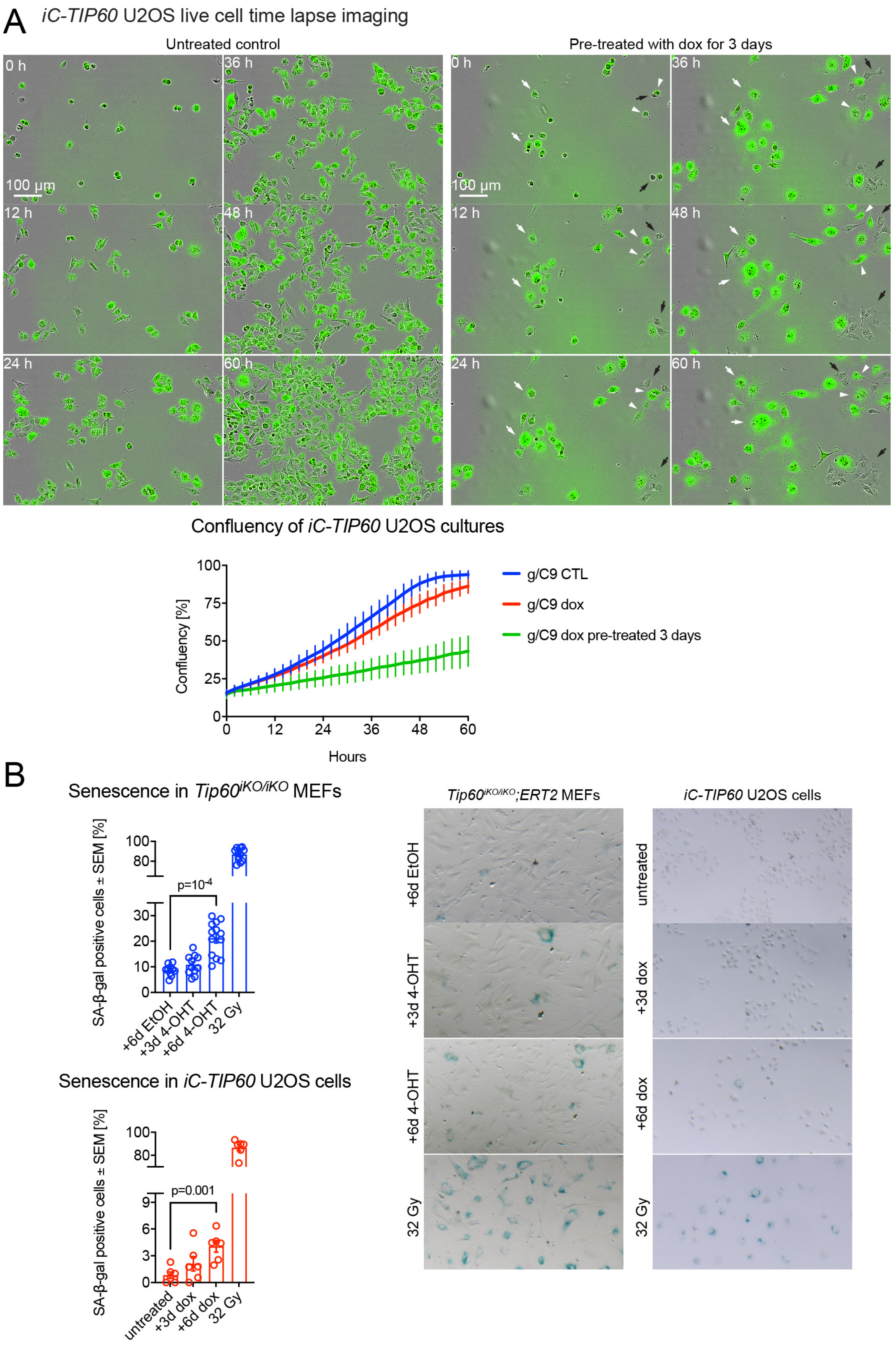

Figure S3. Live-cell time-lapse imaging of *iC-TIP60* U2OS cells.

(A) Representative still images of live-cell time-lapse imaging of *iC-TIP60* and control U2OS after 3 days of pre-treatment with dox and quantification of confluency. The majority of cells did not divide (white arrows) during the 60-h time-lapse imaging period, some cells divided once (white

arrowheads), and some displayed normal division rates (black arrows). No detaching or disintegrating cells were observed, indicating absence of cell death. Automatic image analysis with the IncuCyte S3 Software (V2018B) was used to determine the level of confluency of cells (lower panel), determined by the space occupied by cells compared to total imaged area. Means of 4 images for each of 4 experiments were analyzed.

(B) SA-β-galactosidase (β-gal) staining on *iC-TIP60* U2OS cells and *TIP60*$^{iKO/iKO}$*;ERT2* MEFs 3 and 6 days after *TIP60* deletion. Cells irradiated with 32 Gy were stained as positive controls. Means of β-gal positive cell populations for at least 2 images of 3 replicates with at least 200 counted cells per replicate are displayed and were analyzed by unpaired two-tailed t-test. Circles represent individual datapoints of replicates (B).

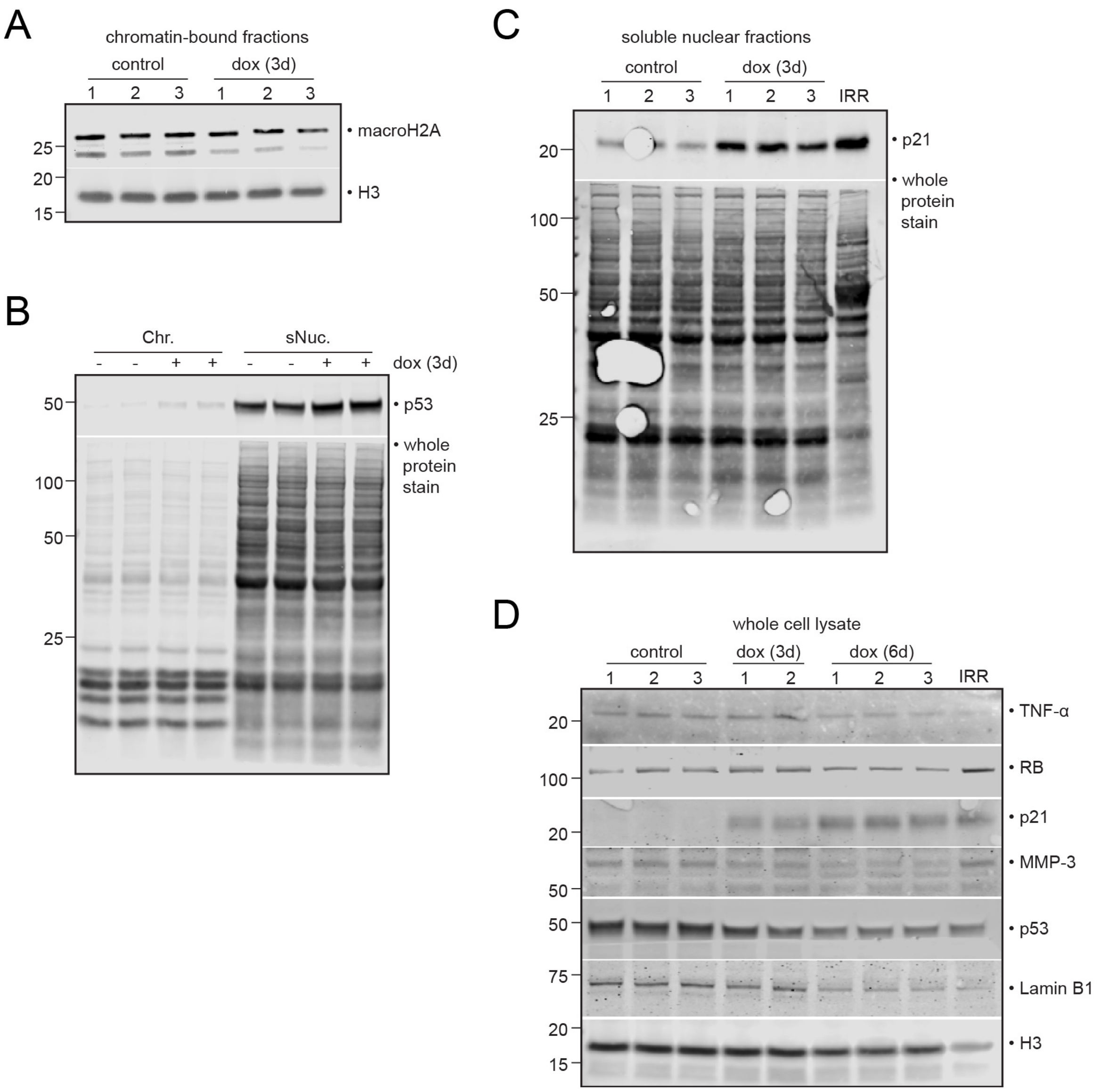

Figure S4. Effects of loss of TIP60 on senescence and senescence-associated marker proteins. Senescence and senescence-associated secretory phenotype (SASP) marker assessed by Western blot in *iC-TIP60* U2OS cells 3 or 6 days after dox treatment as indicated.

(A) MacroH2A in chromatin-bound fractions remains unchanged, indicating the lack of senescence associated heterochromatin.

(B) p53 protein levels in chromatin-bound and soluble nuclear fractions are slightly increased.

(C) p21 protein levels in soluble nuclear fractions are increased.

(D) SASP marker in whole cell lysates. The positive control of irradiated U2OS cells (32 Gy) show decreased H3 levels, which were used as a loading control. Similarly, cells incubated for 6 days in dox-supplemented medium show slightly reduced H3 levels.

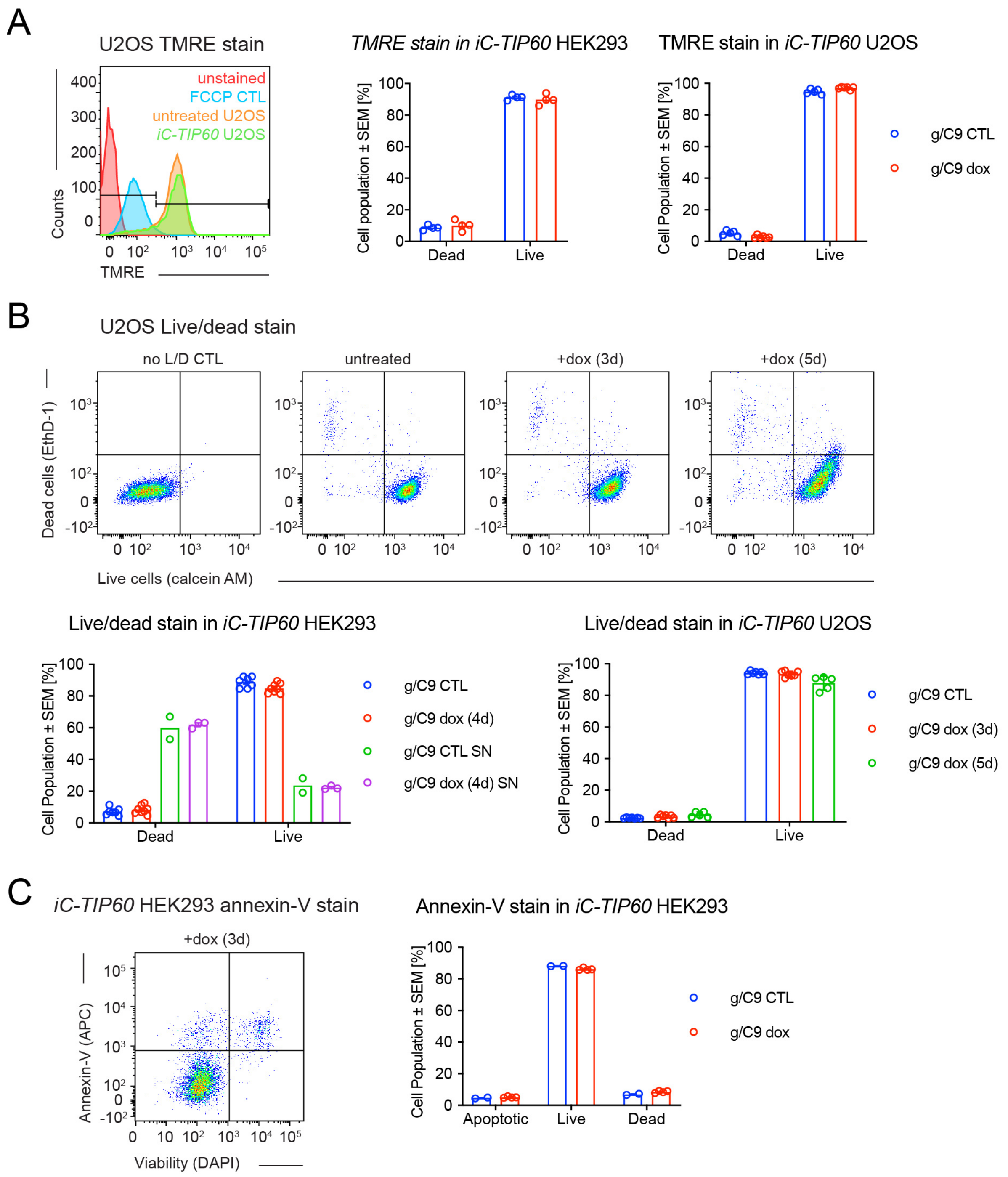

Figure S5. Loss of TIP60 does not lead to increased apoptosis.

(A) *iC-TIP60* HEK293 and *iC-TIP60* U2OS cells and their respective controls were stained with tetramethylrhodamine, ethyl ester (TMRE) and analyzed by flow cytometry. TMRE stains intact, active, negatively charged mitochondria. Apoptotic processes lead to loss of TMRE staining. No change in TMRE staining was observed after loss of TIP60 in HEK293 cells or U2OS cells. Mitochondria depolarized with carbonyl cyanide 4-(trifluoromethoxy) phenylhydrazone (FCCP) were used as a negative control for TMRE staining.

(B) *iC-TIP60* HEK293 and *iC-TIP60* U2OS cells and their respective controls were stained with ethidium homodimer-1 (EthD-1) and calcein AM (calcein [(acetyloxy)methyl ester]) and analyzed

by flow cytometry to provide a second assessment of live and dead cell populations. No change in EtD-1/calcein AM staining was observed after loss of TIP60 in HEK293 cells or U2OS cells. Due to the increase in detached cells in *iC-TIP60* HEK293 cultures, cells in the culture supernatant (SN) were also analyzed.

(C) *iC-TIP60* and control HEK293 cells were stained with annexin V to provide a third assessment of cell death and assessment of apoptotic cell death. No change in annexin V staining was observed after loss of TIP60 in HEK293 cells.

Means ± SEM of 2-8 independent replicates analyzed by unpaired two-tailed t test are displayed (A-C). Circles represent individual datapoints of replicates (A-C)).

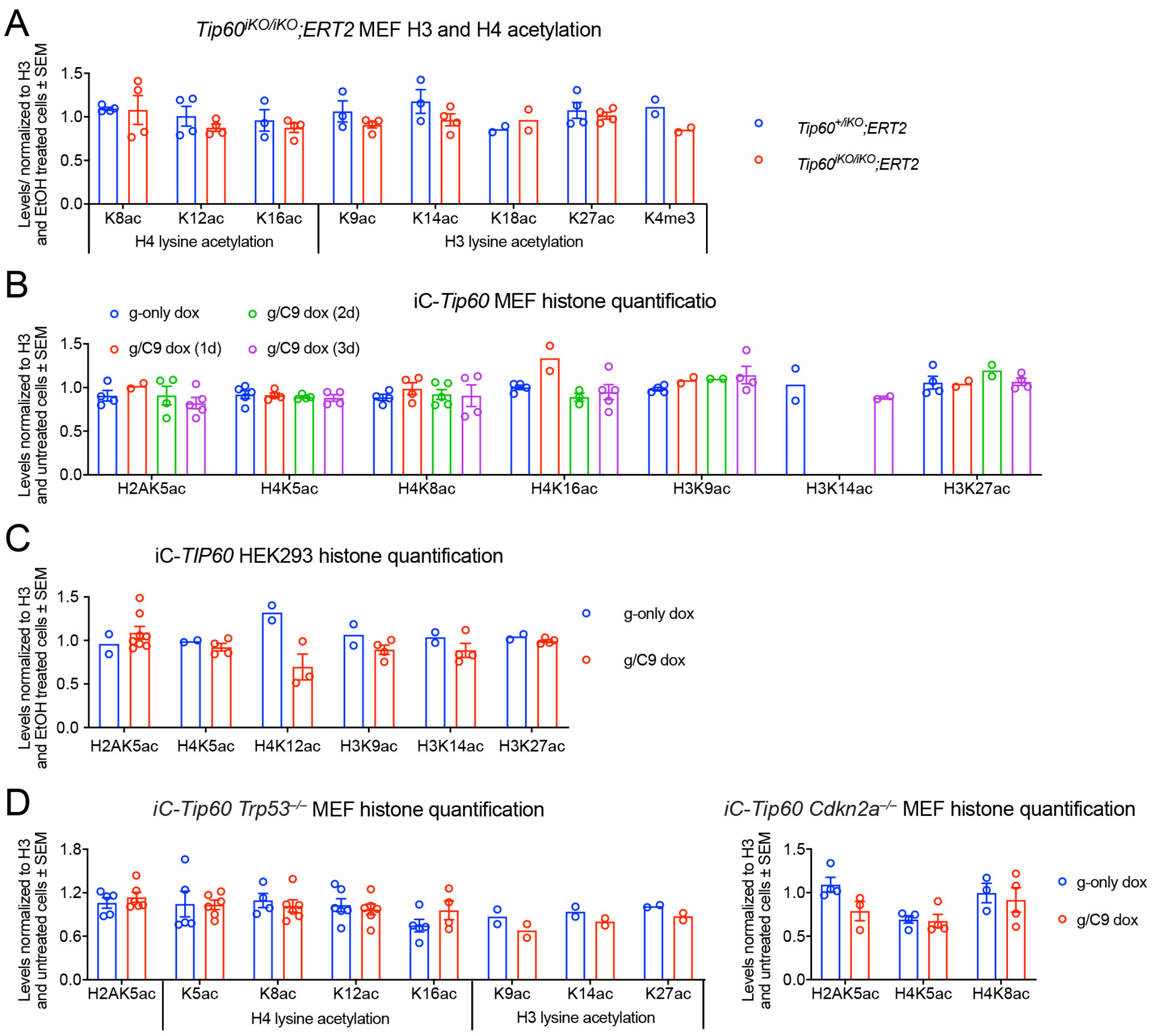

Figure S6. Quantification of lysine acetylation levels on histones H2A, H3 and H4.

Histone acetylation and total protein levels were assessed by Western blotting follwed by densitometry.

(A) Relative fold change in histone lysine acetylation levels normalized to total H3 protein in EtOH and 4-OHT treated *Tip60*$^{fl/+}$*;ERT2*, and *Tip60*$^{fl/fl}$*;ERT2* MEFs, resulting in *Tip6*$^{iKO/+}$*;ERT2* and *Tip60*$^{iKO/iKO}$*;ERT2* MEFs, after 3 days of 4-OHT treatment.

(B) Fold change in histone lysine acetylation levels normalized to H3 in dox treated *iC-Tip60* MEFs and dox treated sgRNA controls MEFs (g-only).

(C) Fold change in histone lysine acetylation levels normalized to H3 between dox treated *iC-TIP60* HEK293 and g-only HEK293 cells, (H4K12ac did not reach statistical significance).

(D) Fold change in histone lysine acetylation levels normalized to H3 between *iC-TIP60 Trp53*$^{-/-}$ MEFs and *iC-TIP60 Cdkn2*$^{-/-}$ MEFs with their respective dox treated g-only controls, 3 days after treatment.

Means ± SEM of 2 to 8 independent experiments are shown. Fold changes were log transformed and analyzed by one-way ANOVA with Benjamini and Hochberg correction (B) or unpaired two-tailed t test (A, C, and D). Circles represent individual datapoints of replicates.

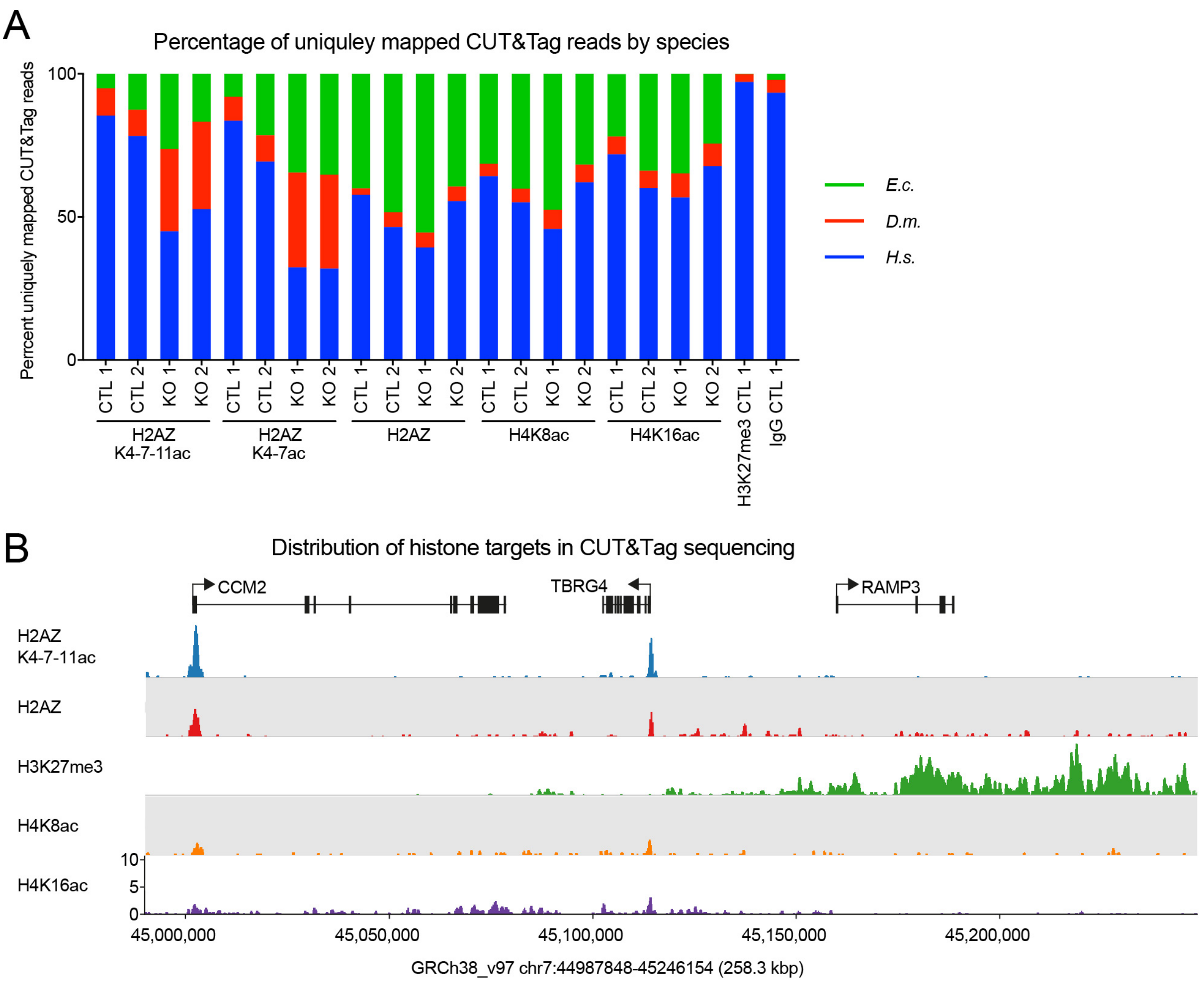

Figure S7. CUT&Tag sequencing comparing histone H2AZ, H2AZK4-7-11ac, H4K8ac, and H4K16ac in *iC-TIP60* U2OS and control cells.

(A) CUT&Tag sequencing read percentages uniquely mapped to the *E. coli (E.c.)*, *D. melanogaster (D.m.)*, or *H. sapiens (H.s.)* genomes. *D. melanogaster* material served as a spike-in, while *E. coli* material is carried over with pAG-Tn5 as a contaminant. CUT&Tag sequencing for H3K27me3 and IgG CTL samples were performed with commercial pAG-Tn5, which contains very little carry-over *E.coli* DNA, while other samples were performed with pAG-Tn5 kindly provided by S. Henikoff and contained *E.coli* DNA. The *E.coli* DNA levels were previously used to indicate the ratio between added pAG-Tn5 and enriched genomic fragments from other species (2).

(B) Read depth plot of a 250 kb genomic region. Shown are CUT&Tag reads for H2AZK4-7-11ac, H2AZ, H3K27me3, H4K8ac, and H4K16ac. H3K27me3 is commonly used as a positive control in CUT&Tag sequencing and is found to occupy transcriptionally inactive regions. The IgG control sample resulted in no comparable read counts. H2AZK4-7-11ac, H2AZ, and H4K8ac were found

enriched at transcription start sites of genes. H4K16ac was more broadly distributed in regions negative or low for H3K27me3.

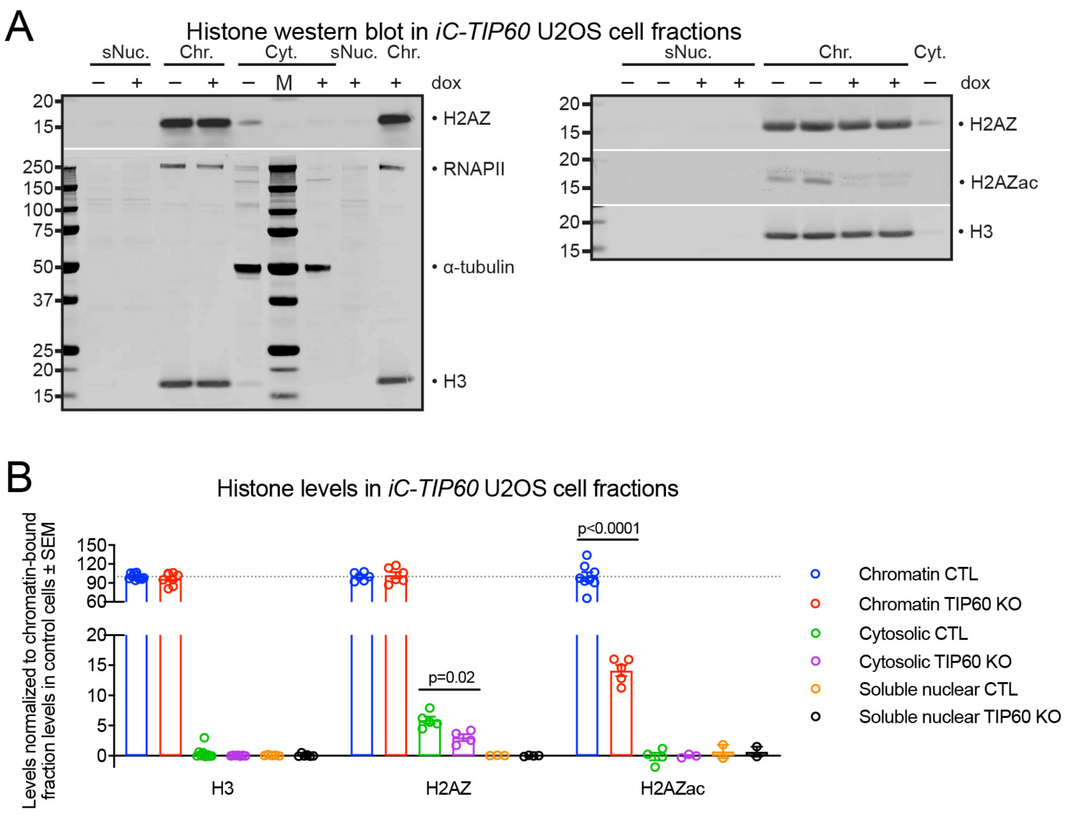

Figure S8. Histone quantification in *TIP60* knockout cell fractions

(A) Western blot of subcellular fractions of *iC-TIP60* U2OS cells 3 days after doxycycline induction and untreated control cells detecting total H3, total H2AZ and H2AZK4-7-11ac. α-tubulin and RNAPII were used as markers for the cytosolic and chromatin-bound fractions, respectively.

(B) Quantification of H3, H2AZ and H2AZK4-7-11ac levels in the subcellular fractions of *iC-TIP60* U2OS cells and control cells, normalized to chromatin-bound fractions in untreated control (CTL) cells. Means ± SEM of 2-6 independent experiments are shown. Fold changes were log transformed and analyzed by unpaired two-tailed t test.

Full-size Western blots for (B) are displayed in the supplemental information.

Circles represent individual datapoints of replicates (B).

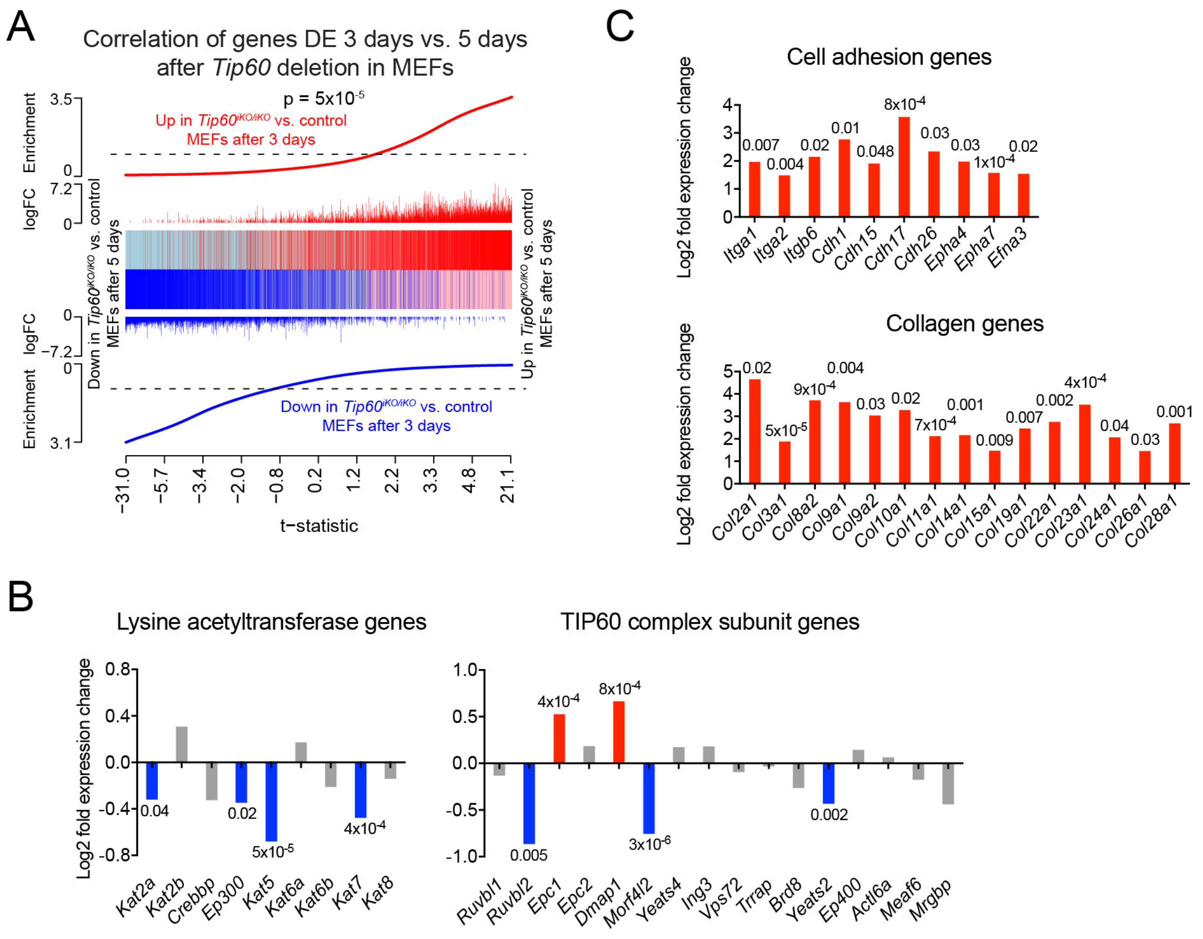

Figure S9. TIP60 deletion in MEFs results in upregulation of cell-substrate adhesion genes.

(A-C) RNA-sequencing results of *Tip60*$^{iKO/iKO}$*;ERT2* vs. *Tip60*$^{+/+}$*;ERT2* MEFs treated with 4-OHT for 3 and 5 days. N = cell isolates from 3 and 4 individual embryos per genotype on days 3 and 5, respectively.

(A) Barcode enrichment plot showing correlation of the transcriptional profiles induced by *Tip60* deletion after 3 days or 5 days in MEFs. Genes are ordered according to differential expression at 5 days from most downregulated (left) to most upregulated (right) in *Tip60* deleted vs. control cells. The x-axis shows moderated t-statistics. Vertical red and blue bars show genes that are differentially expressed 3 days post *Tip60* deletion and the corresponding log2-fold-changes are also shown. The blue and red worms indicate relative enrichment of vertical bars. The ROAST p-value for positive correlation is indicated. A high similarity in the effects of loss of TIP60 at 3 days compared to 5 days after induction of *Tip60* deletion with 4-OHT treatment was observed.

(B, C) Results for specific groups of genes with FDR indicated in the graphs. Blue bars, significantly downregulated, red bars significantly upregulated.

(B) Histone lysine acetyltransferase (KAT) genes and TIP60 complex (NuA4 complex) subunit genes.

(C) Cell-substrate adhesion genes and collagen genes.

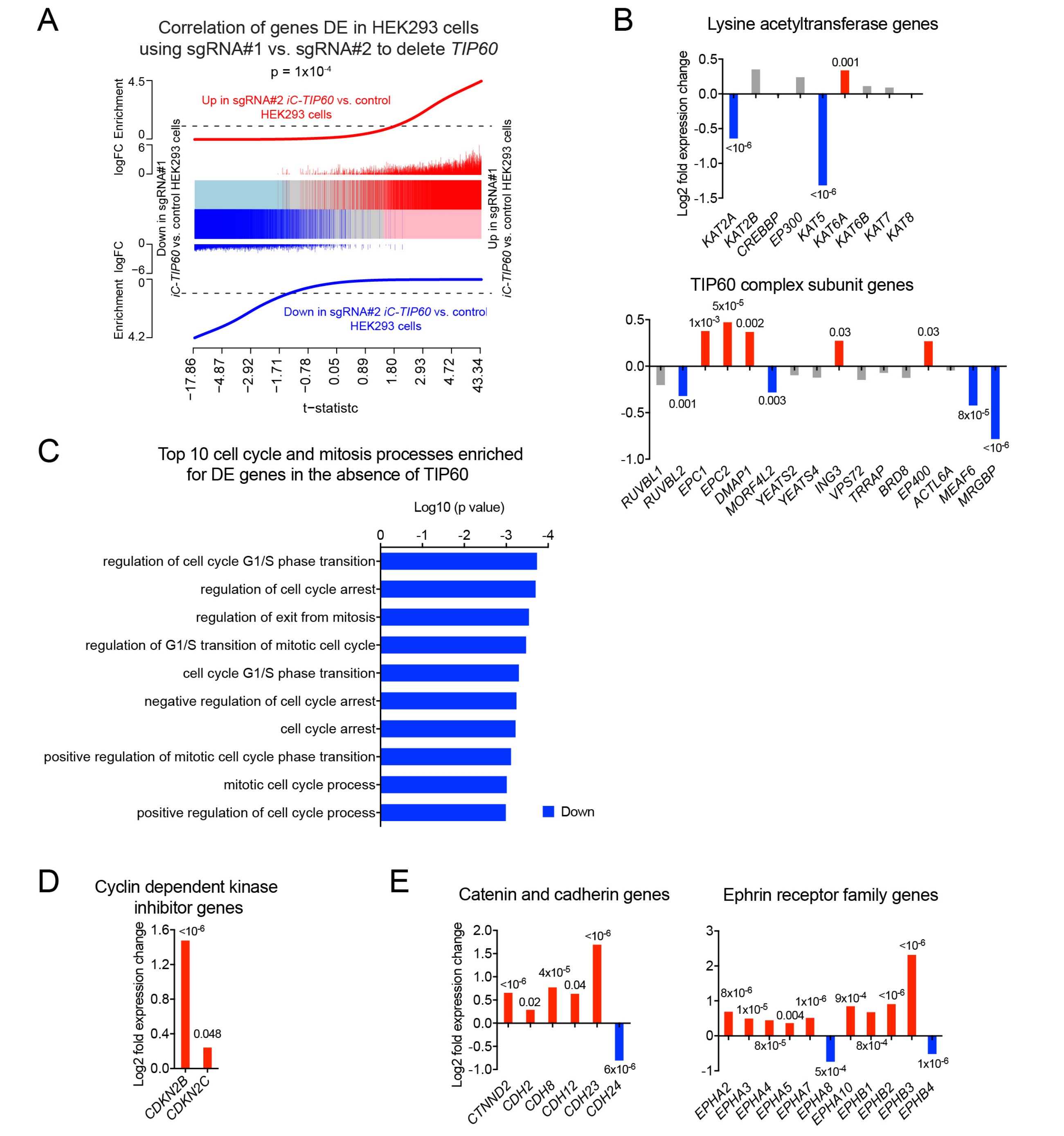

Figure S10. Loss of TIP60 in HEK293 cells results in upregulation of cell-cell adhesion genes. (A-E) RNA-sequencing results comparing *iC-TIP60* vs. control HEK293 cells. Two different sgRNAs were used. HEK293g/C9 after treatment with dox for 3 days were compared to untreated cells (expressing Cas9 only). N = 2 replicate cultures each for sgRNA#1 and sgRNA#2, 4 in total per genotype.

(A) Barcode enrichment plot showing the correlation of the transcriptional profiles induced by *TIP60* deletion after 3 days with either sgRNA#1 or sgRNA#2 in HEK293 cells. Genes are ordered according to differential expression induced with sgRNA#1 from most down-regulated (left) to most up-regulated (right) in *TIP60* deleted vs. control cells. The x-axis shows moderated t-statistics.

Vertical red and blue bars show genes that are differentially expressed after sgRNA#2 induced *TIP60* deletion and the corresponding log2-fold-changes are also shown. The blue and red worms indicate relative enrichment of vertical bars. The ROAST p-value for positive correlation is indicated. The effects of *TIP60* mutation using the two different sgRNAs were highly similar. To focus on effects supported by both sgRNAs, both datasets were combined for further analyses.

(B, D-E) Results for specific groups of genes with FDR. Grey bars represent genes not significantly affected based on FDRs.

(B) Histone lysine acetyltransferase (KAT) genes and TIP60 complex (NuA4 complex) subunit genes.

(C) The top 10 cell cycle and mitosis processes (GO terms) were downregulated in *iC-TIP60* HEK293 cells compared to control cells.

(D) Cyclin dependent kinase inhibitor genes *CDKN2B* and *CDKN2C*.

(E) Cell-cell adhesion genes.

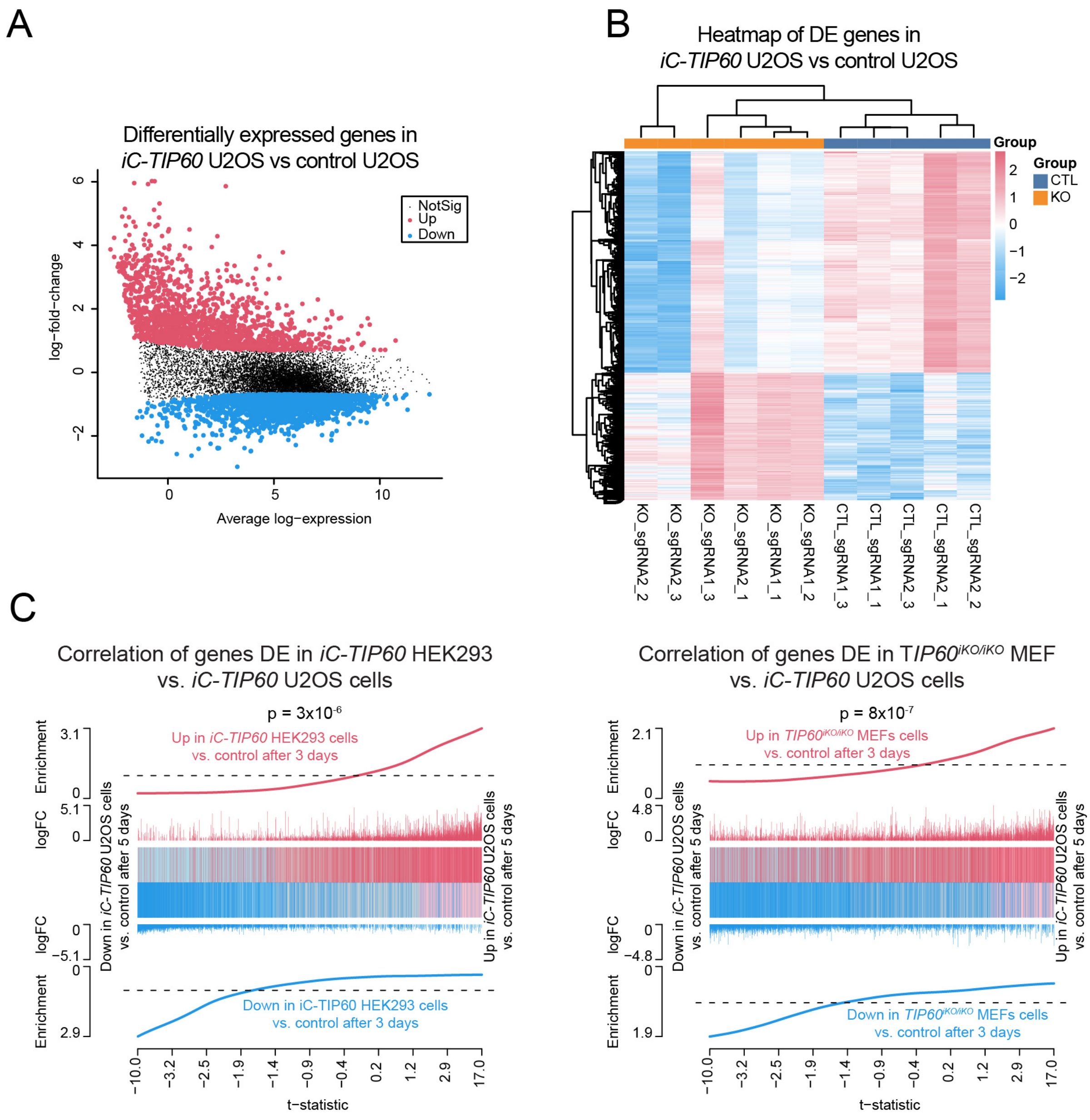

Figure S11. TIP60 is required for normal gene expression in human U2OS cells.

(A-C) RNA-seq results of *iC-TIP60* vs. control U2OS cells after 4 days of dox treatment to induced *Tip60* deletion (n = 6 *iC-TIP60* and 5 control U2OS cell lines).

(A) Mean-difference plot showing the log2-fold change vs. average log2 of counts-per-million of each mRNA in *iC-TIP60* vs. control U2OS cells.

(B) Heatmap of differentially expressed genes in *iC-TIP60* vs. control U2OS cells.

(C) Barcode enrichment plot showing the correlation of the transcriptional profiles induced by *TIP60* deletion after 4 days in U2OS cells compared to HEK293 cells (left panel) and MEFs (right panel). Genes are ordered according to differential expression from most down-regulated (left) to most up-regulated (right) in *TIP60* deleted vs. control U2OS cells. The x-axis shows moderated t-statistics. Vertical red and blue bars show genes that are differentially expressed in *iC-TIP60* HEK293 cells

(left panel) or *Tip60*$^{iKO/iKO}$ MEFs (right panel) after induced *TIP60* deletion and the corresponding log2-fold-changes are also shown. The blue and red worms indicate relative enrichment of vertical bars. The FRY p-values for positive correlation are indicated. The effects of *TIP60* mutation on RNA levels using different cell types and species were highly similar.

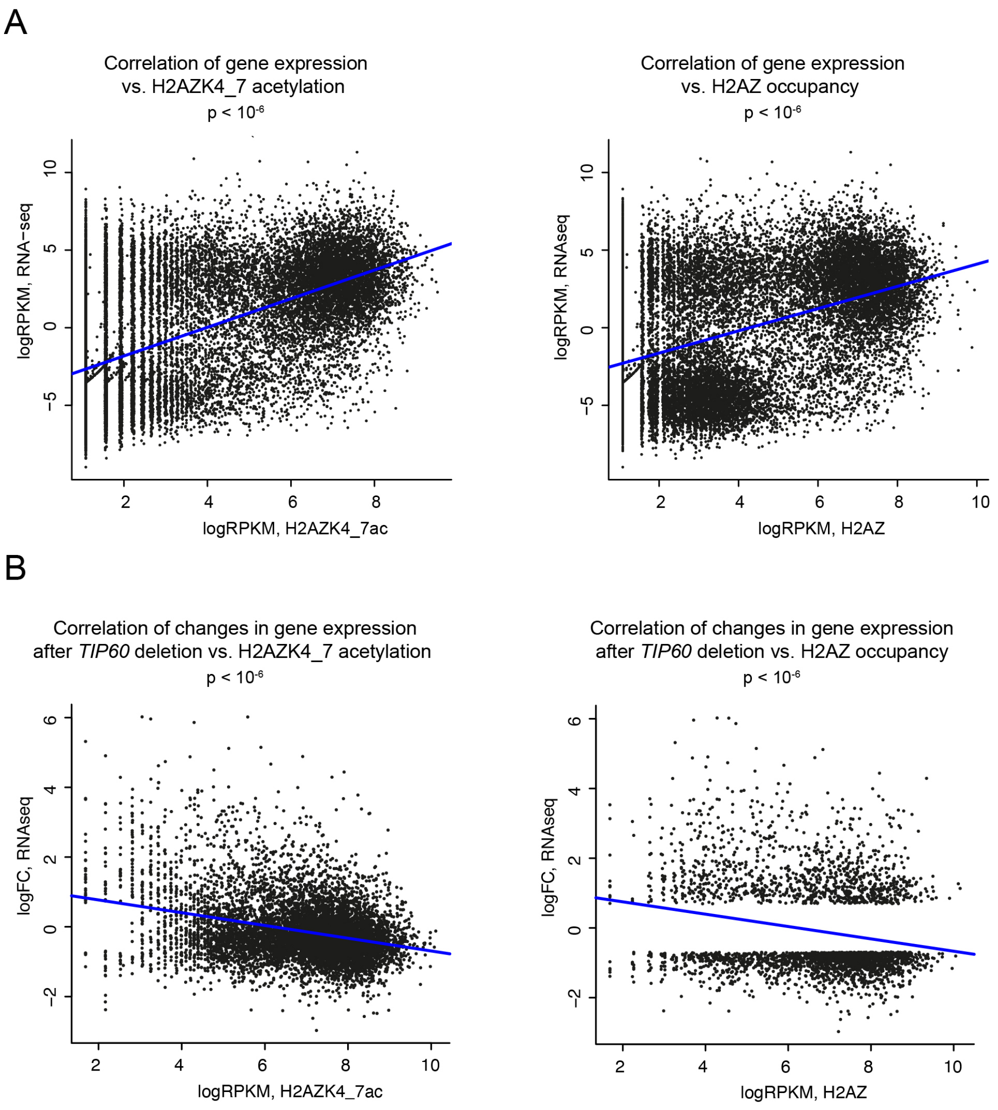

Figure S12. Correlation of H2AZ acetylation and RNA levels.

(A) Correlation between gene expression levels assessed by RNA-seq and H2AZK4_7ac (left panel) or total H2AZ occupancy (right panel) assessed by CUT&Tag in the interval -1 kb to + 1 kb relative to the TSS in control U2OS cells. Each dot represents one gene arranged its RNA level (y-axis) and H2AZK4_7ac (left panel) or total H2AZ occupancy (right panel) assessed by CUT&Tag (x-axis). All genes were included.

(B) Correlation between fold-changes in RNA levels in *iC-TIP60* vs. control U2OS cells assessed by RNA-seq and H2AZK4_7ac (left panel) or total H2AZ occupancy (right panel) assessed by CUT&Tag in the interval -1 kb to + 1 kb relative to the TSS in control U2OS cells. Each dot represents one gene arranged its fold-change in RNA levels (y-axis) and H2AZK4_7ac (left panel) or total H2AZ occupancy (right panel) assessed by CUT&Tag in control U2OS cells (x-axis). Only genes with differentially expressed RNA levels and/or differential H2AZac or total H2AZ were included.

Supplementary Data Tables

Table S1. Number of differentially expressed genes (FDR < 0.05; RefSeq gene annotation) in *Tip60$^{iKO/iKO}$;ERT2* MEF vs. *Tip60$^{+/+}$;ERT2* after 3 days and 5 days of 4-OHT induction and in *iC-TIP60* vs. control HEK293 cells.

	**KO vs. C* MEFs 3D**	**KO vs. C MEFs 5D**	**KO vs. C MEFs 3D and 5D combined**	**C MEFs 5D vs. 3D**	**KO MEFs 5D vs. 3D**	**KO vs. C HEK293 cells**
**Total**	6311	7583	8238	5521	2374	6236
**Down**	3428	3747	4162	3032	1292	2940
**Not Sig.**	7877	6605	5950	8667	11814	8217
**Up**	2883	3836	4076	2489	1082	3296

*KO, knockout vs. C, control.

Supplementary Tables S2 to S9 are supplied as Excel files:

Table S2. Genes differentially expressed in *Tip60$^{iKO/iKO}$;ERT2* MEF vs. *Tip60$^{+/+}$;ERT2* MEFs 3 days after tamoxifen treatment

Table S3. GO term BP in *Tip60$^{iKO/iKO}$;ERT2* MEF vs. *Tip60$^{+/+}$;ERT2* MEFs 3 days after tamoxifen treatment

Table S4. KEGG pathway in *Tip60$^{iKO/iKO}$;ERT2* MEF vs. *Tip60$^{+/+}$;ERT2* MEFs 3 days after tamoxifen treatment

Table S5. Genes differentially expressed in *iC-TIP60* vs. control HEK293 cells

Table S6. GO term BP in *iC-TIP60* vs. control HEK293 cells

Table S7. KEGG pathway in *iC-TIP60* vs. control HEK293 cells

Table S8 Genes DE in iC-TIP60 vs control U2OS cells 4 days after dox treatment

Table S9 Comparison of RNA-seq and CUTnTag datat in iC-TIP60 vs. control U2OS cells

Legends for Supplementary Movies

Movies 1A, 1B, 1C, 2A, 2B supplied separately as AVI files

Movie 1. Live cell time-lapse imaging of *iC-TIP60* U2OS cells. Brightfield and GFP fluorescence overlay of *iC-TIP60* U2OS cells that were (A) untreated (expressing Cas9 only), (B) freshly treated with dox to induce the expression of the sgRNA to delete *TIP60*, and (C) pre-treated with dox for 3 days prior to the start of time-lapse imaging. Images were taken with the IncuCyte S3 system.

Movie 2. 3D confocal live cell time-lapse imaging of *iC-TIP60* U2OS cells. Brightfield, GFP fluorescence and SiR-DNA stain overlay of *iC-TIP60* U2OS cells that were
(A) untreated (expressing Cas9 only) and (B) pre-treated with dox to delete TIP60 for 3 days prior to the start of time-lapse imaging. Cells were imaged in 5-minute intervals for 20 h. on a Leica SP8 confocal microscope.

Supplementary Material and Methods

Sample size selection, randomisation and cell line status

The samples sizes are stated in each figure legend. The sample size was chosen in the following manner: (1) The Australian code of practice for the care and use of animals for scientific purposes was taken into consideration, which compels us to use the smallest number of animals required to detect significant differences between experimental groups. This meant that a p value of less than 0.05 needed to be deemed acceptable and the expectation for the detection sensitivity needed to be moderate (a 25% difference). (2) A power calculation was conducted that made the following assumptions based on previous studies of a similar kind:

(a) The biological samples would generally fall within 10% of the mean.

(b) A p value of < 0.05 would be acceptable.

(c) A power of 0.8 would be considered desirable.

(d) A difference of 25% in the mean should be detectable.

With these input parameters, the minimum required number of animals per treatment group is at least 3. Cells were isolated from mouse embryos in the order as they were obtained from timed matings. Three isolates of each required genotype were used for the experiments without prior knowledge of their performance. Individual human cell lines were obtained on only one occasion and then used without further selection. Cell lines were allocated to experimental groups based on genotype (TIP60 deleted or mutated vs. wild-type). No sample was excluded.

Mouse cell lines were isolated from mouse embryos and tested for mycoplasma. Verified human cell lines were obtained from CellBank Australia and tested for mycoplasm.

PCR Genotyping

Genotyping primers are displayed in Methods Table 1. Murine *Tip60* wild-type, floxed, and deleted alleles were identified by three-way PCR using *Tip60* wild-type forward, reverse and *Tip60* KO

specific forward primers, resulting in 196 bp, 236 bp, and 150 bp long fragments, respectively. *Rosa26CreERT2* forward and reverse primers were used to identify *Cre*, resulting in a 500 bp long fragment. *Trp53*$^{-/+}$ mice were genotyped by three-way PCR using *Trp53* wild-type forward, reverse, and *Trp53* KO specific forward primers. *Cdkn2a*$^{-/+}$ mice were genotyped by three-way PCR using *Cdkn2a* wild-type forward, reverse, and *Cdkn2a* KO specific forward primers.

DNA for genotyping was isolated using DirectPCR (Tail) lysis buffer (Viagen, 102-T) according to manufacturer's instructions with 0.4 mg/ml Proteinase K (Roche, 02115828001). 1 µl crude DNA lysate, 10 µl MyTaqTM HS Red Mix (Bioline, BIO-25048), and 9 µl MQ-H2O were used in PCR reactions, run with standard PCR settings (10 min at 94°C followed by 33 cycles of 30 s at 94°C, 30 s at 60°C and 1 min at 72°C, and a final 10 min at 72°C) in a S1000 Thermal Cycler (Biorad), and analyzed using 3% agarose gels.

Acid histone extraction and quantification

Histones were acid extracted, as described earlier (3) with slight modifications. All reagents used are chilled on ice and samples are kept on ice throughout the procedure. Adherent cells were washed twice with PBS, nuclei were extracted for 5 min on ice in nuclear isolation wash buffer (15 mM Tris-HCl, 60 mM KCl, 15 mM NaCl, 5 mM $MgCl_2$, 1 mM $CaCl_2$, and 250 mM sucrose, pH 7.5 (HCl-adjusted), freshly supplemented 1 protease inhibitor cocktail tablet (PIC, Roche, 11697498001) per 10 ml, 1 mM PMSF (Roche, 10837091001), 1 mM DTT (Supelco, 646563), and 5 mM sodium butyrate (Sigma, 303410)) supplemented with 0.2% (v/v) NP-40 Alternative (Millipore, 492016), scraped from the plate, collected, centrifuged for 5 min at 2500x *g* and 4°C, washed trice with nuclear isolation wash buffer (without NP-40 Alternative), lysed for 2 h on ice in 100 µl 0.4 N H_2SO_4 (Sigma, 339741) per $5x10^6$ cells, and centrifuged for 10 min at 10,000x *g*. Nuclear protein lysate supernatants were transferred to new tubes, centrifuged for 5 min at 10,000x *g*, transferred to another new tube with 0.5 volumes of 100% TCA (Sigma, T0699) to achieve 33% (v/v) final concentration of TCA in the nuclear protein lysates. The tubes were inverted several times and incubated for 2 h or overnight

on ice. Precipitated acid extracted histones were collected by centrifugation for 10 min at 10,000x *g*, washed once with 1 ml acidic acetone (0.1% (v/v) HCl in acetone), spun down for 10 min at 17,300x *g*, washed trice with 100% acetone, air dried for 5 to 15 min, resuspended in 100 µl sterile MQ-H_2O per $5x10^6$ cells, spun down for 2 min at 10,000x *g*, transferred to a new tube, aliquoted and snap frozen at -80°C. Protein was quantified using a Bradford assay according to manufacturer's instruction (Biorad, 5000204).

Cell fractionation protein extraction

Cells were fractioned using the subcellular protein fractionation kit for cultured cells (Thermo Scientific, 78840) according to manufacturer's instructions. All buffers were freshly supplemented with 1 protease inhibitor cocktail tablet (PIC, Roche, 11697498001) per 10 ml, 1 mM PMSF (Roche, 10837091001), 1 mM DTT (Supelco, 646563), and 5 mM sodium butyrate (Sigma, 303410). Briefly, cells were washed twice with ice cold PBS, scraped in ice cold PBS, resuspended in 10 volumes of cytoplasmic extraction buffer (CEB) and incubated on ice for 10 min with gentle mixing. Nuclei were spun down for 5 min at 3,000x *g*, the supernatant was collected as the cytosolic fraction and stored at -80°C. Nuclei were resuspended in 10 volumes of membrane extraction buffer (MEB), vortexed for 5 sec and incubated for 10 min with gentle mixing. Nuclei were collected for 5 min at 3,000x *g* and washed with another 5 volumes of MEB, resuspended in 5 volumes of nuclear extraction buffer (NEB), vortexed for 15 sec and incubated at 4°C for 30 min with gentle mixing. The lysate was centrifuged for 5 min at 5,000x *g*, the supernatant was collected as the soluble nuclear fraction and stored at -80°C. The pellet was washed with another 5 volumes of NEB and resuspended in chromatin-bound extraction buffer (NEB supplemented with 5 mM CaCl2 and 3,000 U/ml micrococcal nuclease (MNase)), pipetted 10 times to break up pellet, vortexed for 15 sec, incubated for 5 min at 37°C, vortexed again for 15 sec and spun down for 5 min at 16,000x *g*. The supernatant was collected as the chromatin fraction and stored at -80°C. A follow-up step to extract nuclear

cytoskeletal proteins was omitted. Protein was quantified using a Bradford assay according to manufacturer's instruction (Biorad, 5000204).

SDS-PAGE and immunoblotting

1 to 5 µg of protein extracts were prepared per lane with 2X SDS sample buffer (0.08 M Tris-HCl, pH 6.8, 2.5% (w/v) sodium dodecyl sulfate, 8% (v/v) glycerol, 4 mM β-mercaptoethanol, 0.02% (w/v) bromophenol blue), incubated for 5 min at 95°C, separated by SDS-PAGE using 4-12% (w/v) Bis-Tris polyacrylamide gels (Invitrogen, NP0321BOX) at 110 V in MES running buffer (NP0002, Invitrogen), and transferred onto hydrophobic PVDF membranes (MERCK, IPFL00010) using transfer equipment (BioRad, 170-3930) in Western transfer buffer (150 mM glycine, 20 mM Tris, 20% (v/v) methanol in MQ-H_2O) at 90 V for 1.5 h. Membranes were blocked in Odyssey Blocking Solution (Licor, 927-40000), for 1 h at 4°C, incubated with primary antibodies (Methods Table 3) overnight in Odyssey Blocking Solution supplemented with 0.1% Tween-20 at 4°C, washed in PBST (PBS, 0.1% (v/v) Tween-20) (5 x 5 min), incubated with 1:8000 anti-rabbit-IRDye-800CW (Licor, 925-32211) and 1:16000 anti-mouse-IRDye-680LT (Licor, 925-68020) IgG secondary antibody in Odyssey Blocking Solution supplemented with 0.1% Tween-20 at RT for 1 h, and washed in PBST (5 x 5 min), washed in PBS (5 x 5 min). Protein bands were detected using the Odyssey CLx Imaging System (Licor) and quantified by Odyssey CLx 1.0.18.

RT-qPCR

RT-qPCRs were performed in triplicate. RNA was isolated using RNeasy Kit (Qiagen, 74106) according to manufacturer's instruction with on-column DNase I digestion according to manufacturer's instructions and quantified using a NanoDrop spectrophotometer. 10 µg of extracted RNA were used as a template for the generation of cDNA. Briefly, RNA was incubated with 5 µM Oligo(dT)15 (Promega, C110A), and 10 mM dNTPs (Promega, U1511) for 5 min at 65°C, left on ice for 10 min, added to 5X first-strand buffer (Invitrogen, y02321), containing 10 U/µl Superscript III

(Invitrogen, G19046030), 2 U/µl RNasin (Promega, N2511), and 5 mM DTT (Invitrogen, y00147), incubated at 50°C for 1 h to facilitate reverse transcription of cDNA, and 85°C for 5 min to inactivate residual enzymatic activity and stored at -80°C until required. A 1:4 dilution of cDNA in 9 µl MQ-H_2O, and 10 µl of SYBR sensimix (Bioline, QT605) and 0.25 mM primers (Methods Table 4) were used in qPCR experiments. Triplicates were measured in a 384 LightCycler plate, run with standard PCR settings (10 min at 95°C followed by 45 cycles of 20 sec at 95°C, 30 s at 60°C and 30 sec at 72°C, and a continuous ramp to 95°C) in a LightCycler480 (Roche).

RNA-sequencing

RNA Sequencing was performed on *Tip60*$^{fl/fl}$*;ERT2* and *Tip60*$^{+/+}$*;ERT2* MEFs, 3 replicates per genotype isolated from individual embryos, induced with 4-OHT for 3 days and 5 days, 2 replicate cultures each of HEK293g1/C9, HEK293g2/C9 and guide-only HEK293g1 and HEK293g2 controls (totaling 4 replicates of *TIP60* deleted and 4 replicates of control HEK293 cells) induced with dox for 3 days and 2-3 replicate cultures of U2OSg1/C9, U2OSg2/C9 and guide only U2OSg1 and U2OSg2 controls (totaling 6 replicates of *TIP60* deleted and 5 replicates of control HEK293 cells) were induced with dox for 4 days. RNA was isolated using RNeasy Kit (Qiagen, 74106). *Drosophila* S2 cell total RNA was spiked-in with U2OS total RNA before library preparation. The Stranded TruSeq mRNA Library Kit (Illumina, 20020594) was used to generate barcoded cDNA libraries. The indexed libraries were pooled and diluted to 1.5 pM for paired end sequencing (2x 81 cycles) on a NextSeq 500 instrument using the v2 150 cycle High Output kit (Illumina) as per manufacturer's instructions, generating 80 bp paired-end reads. The base calling and quality scoring were determined using Real-Time Analysis on board software v2.4.6, while the FASTQ file generation and de-multiplexing utilized bcl2fastq conversion software v2.15.0.4.

For *Tip60*$^{fl/fl}$*;ERT2* and *Tip60*$^{+/+}$*;ERT2* MEF samples, all reads were mapped onto the mouse genome, build mm10, using Rsubread's align function, version 1.30.5 (4). The total number of mapped reads per library (readsNumber) are displayed in Methods Table 5. Reads overlapping each Entrez gene

were summarized into counts using featureCounts from Rsubread with inbuilt RefSeq annotation. Differential expression analyses were then undertaken using the edgeR version 3.22.5 (5), and limma version 3.36.5 (6) software packages. Lowly expressed genes were filtered using edgeR's filterByExpr function with default settings. Additionally, obsolete gene IDs, ribosomal RNAs, and sex-linked genes (Xist and all genes unique to the Y-chromosome) were removed. The sex-linked genes were removed to alleviate gender biases. Following filtering, 14,188 genes remained. RNA composition was then normalized using the TMM method (7). Multiple dimensional scaling (MDS) plots were used to determine if substantial variation existed within treatment groups. The distance between each pair of samples is computed as the leading fold change, defined as the root-mean-square of the largest 500 log2 fold changes between that pair of samples. The correlation between samples from the same litter was estimated, the counts were then transformed to log2-counts per million (CPM) with associated precision weights using voom (8). Differential expression between the *Tip60*$^{fl/fl}$*;ERT2* and *Tip60*$^{+/+}$*;ERT2* MEF samples was then assessed using linear models and robust empirical Bayes moderated t-statistics (9). A contrast was also formed to find genes differentially expressed in the KO averaged over 3-day and 5-day treatment periods. The linear models and voom both incorporated the correlation estimate.

For HEK293g1/C9, HEK293g2/C9 and guide-only HEK293g1 and HEK293g2 samples, all reads were mapped using Rsubread's align function to the human genome, build hg38. The total number of reads mapped for each sample is given in Method Table 5. Like the MEF data, gene counts were generated using featureCounts and inbuilt RefSeq annotation, and expression-based filtering was performed using fitlerByExpr with default settings. Additionally, ribosomal RNAs and obsolete gene IDs were also removed. TMM normalization was then applied. Similar to the MEF data, the counts were transformed to log2-CPM with associated precision weights using voom. Differential expression was then assessed between the groups using linear models and empirical Bayes moderated t-statistics.

For each analysis, the p-values were adjusted using the Benjamini and Hochberg method to control the false discovery rate (FDR) below 5%. The multi-dimension scaling (MDS) plots, mean-difference (MD) plot and barcode plots were generated using limma's plotMDS, plotMD and barcodeplot functions respectively. The heatmap was created using the pheatmap software package version 1.0.12. All gene set tests were carried out using the limma functions fry and roast with 9,999 rotations (10). Gene ontology analyses were performed using limma's goana function.

U2OS samples were processed as described earlier for HEK293 samples up to the normalization, when U2OS cells were normalized using the *Drosophila* S2 reads, with the exception that the Gencode/Ensembl annotation instead of the RefSeq annotation was used. In order to normalize the data and scale it according to the *Drosophila* content of each sample, a normalisation factor was calculated in the following manner: (1) the total *Drosophila* count for each sample was determined, (2) each total was divided by the total filtered human counts for that sample, (3) the resulting numbers was divided by the product of all values calculated in step (2) to the power of (1 divided by the number of samples).

CUT&Tag-sequencing

We performed CUT&Tag on U2OSg1/C9 and U2OSg2/C9 either treated with doxycycline for 4 days (KO) or untreated (CTL). An input of 100,000 cells per sample was used as described by the Henikoff laboratory (2) with slight modifications. pAG-Tn5 with adapters were generously gifted by the Henikoff laboratory and later ordered commercially (EpiCypher, 15-1017). All buffers were made up fresh. Digitonin stock solution was made up fresh as 5% (w/v) in DMSO (CST, 12611P). 10 µl concanavalin-A beads (Bangs Laboratories, BP531) per sample were washed twice in 10 volumes of binding buffer (20 mM HEPES pH 7.5 (Sigma, 83264), 10 mM KCl (Sigma, 60142), 1 mM $CaCl_2$ (Sigma, 21115), and 1 mM $MnCl_2$ (Sigma, M1787)) and resuspended in 10 µl binding buffer per sample. Cells were washed twice with PBS, scraped in PBS, counted and mixed with spike-in S2 *D. melanogaster* cells in a ratio of 43:57 (Mammalian cells: S2 cells) for a combined cell number of

100,000 cells per sample. Cells were washed twice in 1 ml wash buffer (20 mM HEPES pH 7.5, 150 mM NaCl (Sigma, 71386), 0.5 mM spermidine (Sigma, S0266), and 1 protease inhibitor tablet per 50 ml buffer (Roche, 05056489001)), resuspended in 90 µl wash buffer per sample, and concanavalin-A beads were added dropwise while vortexing on low setting, incubated for 10 min at RT on a roller. The supernatant was removed with a magnetic rack, and cells resuspended in 100 µl ice-cold antibody buffer (wash buffer supplemented with 0.05 % (w/v) digitonin (Merck, 300410), 2 mM EDTA (Invitrogen, 15575020), and 0.1 % (w/v) BSA (Sigma, A8577)) per sample and split into 50 µl aliquots. Primary antibody was added to respective tubes as indicated in Methods Table 3 and incubated for 2 h, at RT on a roller. The supernatant was removed, 100 µl ice cold dig-wash buffer (wash buffer supplemented with 0.05% (w/v) digitonin) containing secondary antibody as indicated in Methods Table 3 was added to the cells, incubated for 60 min at RT on a roller, washed trice in 1 ml dig-wash buffer, resuspend in 100 µl ice-cold dig-300 buffer (20 mM HEPES pH 7.5, 300 mM NaCl, 0.5 mM spermidine, 0.01 % (w/v) digitonin, and 1 protease inhibitor tablet per 50 ml buffer) supplemented with 1:250 pA-Tn5 adapter complex and incubated for 60 min at RT on a roller. Cells were washed trice in 1 ml dig-300 buffer, resuspended in 100 µl tagmentation buffer (dig-300 buffer supplemented with 0.01 mM $MgCl_2$ (Sigma, 63069)) while gently vortexing and incubated at 37°C for 60 min. Tagmentation was stopped by adding 3.34 µl 0.5 M EDTA, 1 µl 10% (w/v) SDS (Sigma, 71736), and 0.83 µl 20 mg/ml thermolabile proteinase K (NEB, P8111S) to each sample, mixed by vortexing on full speed and incubated at 37°C for 60 min and 800 rpm shaking followed by heat inactivation at 55°C for 10 min. DNA was extracted via Ampure XP beads (Beckman, A63880). Briefly, 122 µl Ampure XP beads were added to each sample and incubated for 5 min at RT, washed twice in 1 ml 80% ethanol without removing from the magnetic rack. All residual ethanol was removed beads were air-dried for 5 min at RT. DNA fragments were eluted in 25 µl TE buffer (10 mM Tris-HCl pH 8.0 (Invitrogen, 15568025), 1 mM EDTA, and 25 µg/ml RNAse A (ThermoFisher, EN0531)) by vortexing and incubation at 37°C for 10 min, and transferred to a new tube. 10 µl sample DNA fragments were used in library PCR mixtures with 100 nM sample specific nextera i5 and i7

primer combinations (11) and 12.5 µl 2X NEBNext Ultra II Q5 Master Mix (NEB, M0541L) in a total of 25 µl and amplified with the following PCR settings: 72°C for 5 min followed by 13 cycles of 98°C for 30 sec, 98°C for 10 sec and 63°C for 10 sec, and 72°C for 1 min. PCR products were cleaned up using 30 µl Ampure XP beads as described above, eluted in 25 µl 10 mM Tris-HCl pH 8.0, and stored at -20°C. The clean libraries were analyzed by High Sensitivity D1000 gels (Agilent, 5067- 5584) on an Agilent 4200 tapestation using 2 µl sample and an electronic ladder. Library concentrations were measured between 125 bp and 1200 bp. Libraries were pooled into a 10 nM library of 20 µl. The pooled indexed libraries were diluted to 1.5 pM for paired end sequencing (2x 36 cycles) on a NextSeq 500 instrument using the v2 150 cycle Mid Output kit (Illumina) as per manufacturer's instructions, generating 36 bp paired-end reads. The base calling and quality scoring were determined using Real-Time Analysis on board software v2.4.6, while the FASTQ file generation and de-multiplexing utilized bcl2fastq conversion software v2.15.0.4.

As the samples contain *H. sapiens*, *D. melanogaster*, and *E. coli* DNA, an index was first built containing all three genomes to allow competitive alignment. This index was built via Rsubread (2.0.1) (4) using builds hg38, R6.35, and K12 substrain MG1655 for the *H. sapiens*, *D. melanogaster* and *E. coli* genomes, respectively. All libraries were aligned to this index using Rsubread's align function with minimum fragment length set to 30. In all cases approximately 90% of fragments (read pairs) mapped uniquely to the combined genomes. The total of uniquely mapped reads per library are displayed in Methods Table 6. Read counts were obtained using Rsubread's featureCounts function. For *D. melanogaster*, read counts were summarized by chromosome. For *H. sapiens*, read counts were obtained for the putative promoter regions (transcription start site (TSS) ± 1000 bp) of protein-coding genes. Gene annotation was obtained from Gencode (hg38 version 35). For genes with multiple TSS, the most 5' start site was used.

Downstream analyses used the limma (3.44.3) (6) and edgeR (3.30.3) (7) software packages. Genes with low counts across all samples were filtered using edgeR's filterByExpr function with default settings. The *H. sapiens* library sizes were normalized by *D. melanogaster* content, i.e., the human

library sizes were then scaled to equalize the total *D. melanogaster* count-per-million across the samples. The resulting normalized library sizes were applied to the human differential occupancy analyses. Biological variation between replicate samples was estimated using edgeR's estimateDisp function. Differential abundance between the *iC-TIP60* and control samples for each histone mark was assessed by generalized linear models and quasi-likelihood F-tests (12). Violin plots were created using ggplot2 (3.3.2). Global tests were conducted using edgeR's fry function (10). Wiggle plots were created using Seqmonk (v1.45.1). Bam file reads were mapped to the human genome (GRCh38.p13) and probes were created in a 2 bp window with 2 bp steps, quantified, and smoothed with a 500 bp smoothing normalisation.

BrdU incorporation, live/death cell assays and flow cytometry

For BrdU incorporation analysis, cells were incubated with 10 µM BrdU for 1 h, trypsinized, washed once with PBS, resuspended in PBS, fixed by dropwise adding -20°C 100% EtOH to a final concentration of 75% EtOH while gently vortexing, and stored at 4°C for up to 7 days. Fixed cells were washed twice with FACS buffer (150 mM NaCl, 3.7 mM KCl, 2.5 mM $CaCl_2$, 1.2 mM $MgSO_4$, 14.8 mM HEPES, 1.2 mM KH_2PO_4, pH 7.2), supplemented with 2% (v/v) FBS (SAFC, 12003C), treated with 2 M HCl for 1 h, washed twice with FACS buffer, stained with 1:20 BrdU antibody (Invitrogen, 17-5071-41) for 30 min, washed twice with FACS buffer, stained with 1:20 7-AAD (BD Biosciences, 559925) and analyzed by flow cytometry.

To identify live cells, cells were treated with 50 nM TMRE (Abcam, ab113852) for 30 min, trypsinized, washed with FACS buffer and analyzed by flow cytometry. Additionally, cells were treated with 100 µM FCCP (Abcam, ab113852) as a negative control 10 min prior to TMRE stain. To quantify live and dead cells, cells were collected, washed twice with PBS, stained in 10 µM EthD-1 and 0.2 µM calcein AM (ThermoFisher, L3224) in PBS for 15 min and analyzed by flow cytometry.

γH2AX staining

For γH2AX staining, cells were trypsinized and washed twice with PBS. $1x10^5$ cells were fixed and permeabilized in 100 µl FOXP3 fixation and permeabilization solution (ThermoFisher, 00-5523-00) according to manufacturer's instruction, incubated for 1 h, washed twice with FACS buffer, resuspended in 500 µl FACS buffer containing 1:400 anti-γH2AX antibody (Merck, 16-193), and incubated overnight at 4°C on a roller. Stained cells were washed twice in FACS buffer and resuspended in 100 µl FACS buffer containing 1:400 streptavidin-A647 conjugate (ThermoFisher, S21374), and incubated for 1 h, at 4°C. Stained cells were washed twice with FACS buffer, resuspended in 200 µl FACS buffer containing 25 µg/ml RNAse (Merck, 10109142001) and 0.5 µg/ml DAPI (Sigma, 10236276001), and analyzed by flow cytometry. All buffers contained phosphatase inhibitor according to manufacturer's instructions (Merck, 4906845001). As a positive control, wild-type MEFs were irradiated with 20 mJ and recovered for 30 min. γH2AX intensities for cells with 2n and 4n DNA content were analyzed separately and normalized to the respective control value.

Methods Tables

Methods Table 1. Genotyping primers.

Primer	Sequence
Tip60 wild-type forward	GGTGAGTAGGTCCCCCATTTC
Tip60 wild-type reverse	ATCCCACCCTCTGCCTTCTCT
Tip60 KO specific forward	GCGGTCGCAGATACTCGG
Rosa26CreERT2 forward	CGCGGTCTGGCAGTAAAAAC
Rosa26CreERT2 reverse	GCAGATGGCGCGGCAACACC
Trp53 wild-type forward	TTATGAGCCACCCGAGGT
Trp53 wild-type reverse	TATACTCAGAGCCGGCCT
Trp53 KO specific forward primer	TCCTCGTGCTTTACGGTATC
Cdkn2a wild-type forward	GTGATCCCTCTACTTTTTCTTCTGACTT
Cdkn2a wild-type reverse	CGGAACGCAAATATCGCAC
Cdkn2a KO specific forward primer	GAGACTAGTGAGACGTGCTACTTCCA

Methods Table 2. NGS primers used to sequence indels in CRISPR mutated cell lines

Target	Fwd primer	Rev primer
Murine sgRNA1 Tip60	GTGACCTATGAACTCAGGAGTCgatggaata ccgtcagcacca	CTGAGACTTGCACATCGCAGCcccaagacccctca taccaag
Murine sgRNA2 Tip60	GTGACCTATGAACTCAGGAGTCgtgggctact tctccaagg	CTGAGACTTGCACATCGCAGCggcaaagtagatgc tggaacct
Murine sgRNA1 POT1*	GTGACCTATGAACTCAGGAGTCgagagcttta aagtgcgggc	CTGAGACTTGCACATCGCAGCgaccaaaggggta ggcgtag
Murine sgRNA1 POT3	GTGACCTATGAACTCAGGAGTCacgctgttttc tggcgtaca	CTGAGACTTGCACATCGCAGCaggatggacagtgc agttgg
Murine sgRNA1 POT4	GTGACCTATGAACTCAGGAGTCaggggcata aagcccaaacg	CTGAGACTTGCACATCGCAGCagtagtcttctgtagt tgttgctct
Murine sgRNA1 POT5	GTGACCTATGAACTCAGGAGTCgttttctgcg cacggagttg	CTGAGACTTGCACATCGCAGCttcacctaccacctc gacct
Murine sgRNA2 POT1	GTGACCTATGAACTCAGGAGTCctggggaga gggtgttattgac	CTGAGACTTGCACATCGCAGCctggcagtggcaga aagaatc
Murine sgRNA2 POT2	GTGACCTATGAACTCAGGAGTCacaggagg ggtgggaatacg	CTGAGACTTGCACATCGCAGCggacactcgggga agagttt
Murine sgRNA2 POT4	GTGACCTATGAACTCAGGAGTCagaggacta ggcgaggtgat	CTGAGACTTGCACATCGCAGCacacagcaatttcca ggagga
Murine sgRNA2 POT5	GTGACCTATGAACTCAGGAGTCttggtccaag actctccagg	CTGAGACTTGCACATCGCAGC ttgacacactggaatgggct
Murine sgRNA2 POT7	GTGACCTATGAATCAGGAGTC tgctttcctacgtacgcact	CTGAGACTTGCACATCGCAGC agtgcgtacgtaggaaagca

*The 7 top potential off-target (POT) loci were also sequenced and no indels were detected.

Methods Table 3. Primary and secondary antibodies used in Western blot (WB), immunofluorescence (IF) and CUT&Tag experiments

Primary antibody	Type	Target	WB	CUT&Tag
Abcam, ab214725	rabbit monoclonal	H2AZK4ac	1:2000	
Abcam, ab214730	rabbit monoclonal	H2AZK7ac	1:1000	
Millipore, ABE1363	rabbit polyclonal	H2AZK4-7-11ac	1:2000	
Abcam, ab4174	rabbit monoclonal	H2AZ	1:750	
Abcam, ab45152	rabbit monoclonal	H2AK5ac	1:4000	
Abcam, ab4729	rabbit monoclonal	H2AK27ac		
Millipore, 07-327	rabbit polyclonal	H4K5ac	1:5000	
Millipore, 07-328	rabbit polyclonal	H4K8ac	1:5000	1:100
Abcam, ab46983	rabbit polyclonal	H4K12ac	1:20000	
Millipore, 07-329	rabbit polyclonal	H4K16ac	1:7500	1:100
Abcam, ab4441	rabbit monoclonal	H3K9ac	1:8000	
Millipore, 07-353	rabbit polyclonal	H3K14ac		
CST, D4B9	rabbit monoclonal	H3K14ac	1:1000	
Abcam, ab1191	rabbit polyclonal	H3K18ac	1:1000	
Abcam, ab4729	rabbit polyclonal	H3K27ac	1:2000	
Millipore, 07-473	rabbit polyclonal	H3K4me3	1:7500	
Millipore, 16-193	Mouse monoclonal, biotin-conjugated	phospho-H2AX		
Diagenode, C15410173	rabbit polyclonal	H2AZK4-7-11ac	1:3000	1:250
CST, D3V1I	rabbit monoclonal	H2AZK4-7ac		1:200
Active Motif, AB_2615081	rabbit polyclonal	H2AZ	1:3000	1:200
Abcam, ab10799	mouse monoclonal	H3	1:7000	
**Secondary antibody or conjugate**	**Type**	**Target**	**WB**	
LiCor, 926-32211	Goat monoclonal	anti-rabbit-IRDye800CW	1:8000	
LiCor, 925-68020	Goat monoclonal	anti-mouse-IRDye680LT	1:16000	
Antibodies online, ABIN101961	Guinea pig polyclonal	α-rabbit IgG		1:100

Methods Table 4. RT-qPCR primers.

Target mRNA	Fwd primer	Rev primer	An. T [°C]
Murine *Tip60*, exon 8	CCACAAGAGCTTACCACGCT	AGGGTGCCGAAGATCACATT	62
Murine *Tip60*, exon 11	GGGCTATGGCAAGCTGCTTATT	GCCAAGATCTGACAGGGGTTT	62
Murine *Tip60*, exon 3, 4	GATGAGTGGCCCCTGGC	GCTCGTGAGTCACCCATTCA	61
Murine *Gapdh*	TTCACCACCATGGAGAAGGC	CCCTTTTGGCTCCACCCT	60
Murine *Hsp90ab1*	ACCTGGGAACCATTGCTAAG	AGAATCCGACACCAAACTGC	60
Murine *Rpl13*	TGAGGACCTCTGTGAACTTGC	GGAGAAACGGAAGGAAAAGG	60
Human Tip60, exon 8	CAACCACCGCTCAACGAAAC	AGAAGTACCACGGCTTGAGG	60
Human Tip60, exon 11	CTAACCCTGCCTCCCTACCA	TGGTCTGGGACCAGTAGCTT	60
Human Gapdh	TGCACCACCAACTGCTTAGC	GGCATGGACTGTGGTCATGAG	60
Human Hsp90ab1	AATTGACATCATCCCCAACCCT	CCAAACTGCCCAATCATGGAG	60

Methods Table 5. Total number of mapped reads per RNA-seq library.

Library	Number of mapped sequencing reads
Tip60$^{iKO/iKO}$;ERT2 MEF 3 days 1 (KO_3D_1)	25966327
Tip60$^{iKO/iKO}$;ERT2 MEF 3 days 2 (KO_3D_2)	44844743
Tip60$^{iKO/iKO}$;ERT2 MEF 3 days 3 (KO_3D_3)	2512975
Tip60$^{iKO/iKO}$;ERT2 MEF 3 days 4 (KO_3D_4)	11942530
Tip60$^{iKO/iKO}$;ERT2 MEF 5 days 1 (KO_5D_1)	31552772
Tip60$^{iKO/iKO}$;ERT2 MEF 5 days 2 (KO_5D_2)	15649991
Tip60$^{iKO/iKO}$;ERT2 MEF 5 days 3 (KO_5D_3)	22524297
Tip60$^{iKO/iKO}$;ERT2 MEF 5 days 4 (KO_5D_4)	18205962
Tip60$^{+/+}$;ERT2 MEF 3 days 1 (C_3D_1)	10586113
Tip60$^{+/+}$;ERT2 MEF 3 days 2 (C_3D_2)	15022993
Tip60$^{+/+}$;ERT2 MEF 3 days 3 (C_3D_3)	12731188
Tip60$^{+/+}$;ERT2 MEF 3 days 4 (C_3D_4)	8554774
Tip60$^{+/+}$;ERT2 MEF 5 days 1 (C_5D_1)	17673369
Tip60$^{+/+}$;ERT2 MEF 5 days 2 (C_5D_2)	22268708
Tip60$^{+/+}$;ERT2 MEF 5 days 3 (C_5D_3)	6987679
Tip60$^{+/+}$;ERT2 MEF 5 days 4 (C_5D_4)	16435287
iC-TIP60 HEK293 sgRNA#1 1 (KO_sgRNA1_1)	19567415
iC-TIP60 HEK293 sgRNA#1 2 (KO_sgRNA1_2)	15411602
iC-TIP60 HEK293 sgRNA#2 1 (KO_sgRNA2_1)	21735884
iC-TIP60 HEK293 sgRNA#2 2 (KO_sgRNA2_2)	17414394
HEK293 sgRNA#1 1 (C_sgRNA1_1)	14557407
HEK293 sgRNA#1 2 (C_sgRNA1_2)	4387017
HEK293 sgRNA#2 1 (C_sgRNA2_1)	16987280
HEK293 sgRNA#2 2 (C_sgRNA2_2)	14425801

Methods Table 6. Total number of mapped reads per CUT&Tag library.

Library	Number uniquely mapped sequencing reads			
	Total	*H. sapiens*	*D. melanogaster*	*E. coli*
iC-TIP60 U2OSg1 control H2AZK4-7-11ac	8504100	7231571	800755	429024
iC-TIP60 U2OSg2 control H2AZK4-7-11ac	8561817	6668753	783104	1066582
iC-TIP60 U2OSg1 KO H2AZK4-7-11ac	6135341	2744741	1760245	1596954
iC-TIP60 U2OSg2 KO H2AZK4-7-11ac	3722353	1949730	1134295	618118
iC-TIP60 U2OSg1 control H2AZK4-7ac	4071760	3390251	337310	325488
iC-TIP60 U2OSg2 control H2AZK4-7ac	4176216	2879607	387024	889721
iC-TIP60 U2OSg1 KO H2AZK4-7ac	2213318	712549	731506	759042
iC-TIP60 U2OSg2 KO H2AZK4-7ac	1964805	624154	643374	688349
iC-TIP60 U2OSg1 control H2AZ	7179687	4125194	165026	2853561
iC-TIP60 U2OSg2 control H2AZ	8506536	3928537	441402	4088764
iC-TIP60 U2OSg1 KO H2AZ	4321923	1693417	220087	2386535
iC-TIP60 U2OSg2 KO H2AZ	6795090	3754220	342894	2658398
iC-TIP60 U2OSg1 control H4K8ac	4567993	2922479	194570	1430454
iC-TIP60 U2OSg2 control H4K8ac	3122626	1713702	147589	1246149
iC-TIP60 U2OSg1 KO H4K8ac	2998455	1367766	199145	1418387
iC-TIP60 U2OSg2 KO H4K8ac	4499754	2782847	277091	1418979
iC-TIP60 U2OSg1 control H4K16ac	8895096	6353215	555080	1932224
iC-TIP60 U2OSg2 control H4K16ac	11031665	6597907	665034	3707213
iC-TIP60 U2OSg1 KO H4K16ac	8188451	4627658	676370	2835401
iC-TIP60 U2OSg2 KO H4K16ac	4349355	2925796	346166	1049673
iC-TIP60 U2OSg1 control H3K27me3	8397551	8110135	235875	26
iC-TIP60 U2OSg1 control IgG	50439	46627	2274	1005

References

1. Hodgkins A, Farne A, Perera S, Grego T, Parry-Smith DJ, Skarnes WC, et al. WGE: a CRISPR database for genome engineering. Bioinformatics. 2015;31(18):3078-80.
2. Kaya-Okur HS, Wu SJ, Codomo CA, Pledger ES, Bryson TD, Henikoff JG, et al. CUT&Tag for efficient epigenomic profiling of small samples and single cells. Nat Commun. 2019;10(1):1930.
3. Sidoli S, Bhanu NV, Karch KR, Wang X, Garcia BA. Complete Workflow for Analysis of Histone Post-translational Modifications Using Bottom-up Mass Spectrometry: From Histone Extraction to Data Analysis. J Vis Exp. 2016(111):e54112.
4. Liao Y, Smyth GK, Shi W. The R package Rsubread is easier, faster, cheaper and better for alignment and quantification of RNA sequencing reads. Nucleic Acids Res. 2019;47(8):e47.
5. McCarthy DJ, Chen Y, Smyth GK. Differential expression analysis of multifactor RNA-Seq experiments with respect to biological variation. Nucleic Acids Res. 2012;40(10):4288-97.
6. Ritchie ME, Phipson B, Wu D, Hu Y, Law CW, Shi W, et al. limma powers differential expression analyses for RNA-sequencing and microarray studies. Nucleic Acids Res. 2015;43(7):e47.
7. Robinson MD, Oshlack A. A scaling normalization method for differential expression analysis of RNA-seq data. Genome Biol. 2010;11(3):R25.
8. Law CW, Chen Y, Shi W, Smyth GK. voom: Precision weights unlock linear model analysis tools for RNA-seq read counts. Genome Biol. 2014;15(2):R29.
9. Phipson B, Lee S, Majewski IJ, Alexander WS, Smyth GK. Robust Hyperparameter Estimation Protects against Hypervariable Genes and Improves Power to Detect Differential Expression. Ann Appl Stat. 2016;10(2):946-63.
10. Wu D, Lim E, Vaillant F, Asselin-Labat ML, Visvader JE, Smyth GK. ROAST: rotation gene set tests for complex microarray experiments. Bioinformatics. 2010;26(17):2176-82.
11. Mezger A, Klemm S, Mann I, Brower K, Mir A, Bostick M, et al. High-throughput chromatin accessibility profiling at single-cell resolution. Nature Communications. 2018;9(1):3647.
12. Lun AT, Chen Y, Smyth GK. It's DE-licious: A Recipe for Differential Expression Analyses of RNA-seq Experiments Using Quasi-Likelihood Methods in edgeR. Methods Mol Biol. 2016;1418:391-416.
