# Supplementary material for: Loss of TIP60 (KAT5) abolishes H2AZ lysine 7 acetylation and causes p53, INK4A, and ARF-independent cell cycle arrest
Source: Cell Death Dis. 2022 Jul 20;13(7):627. doi: 10.1038/s41419-022-05055-6 (PMC9296491; doi:10.1038/s41419-022-05055-6)
Supplement: Supplementary file 15 — Original Data File [file 41419_2022_5055_MOESM15_ESM.pdf]

Trp53$^{-/-}$ and *Cdkn2a*$^{-/-}$ *iC-Tip60* MEFs

Cdkn2a$^{-/-}$ *iC-Tip60* MEFs western blot raw data for Figure 7 E and Figure S4 D

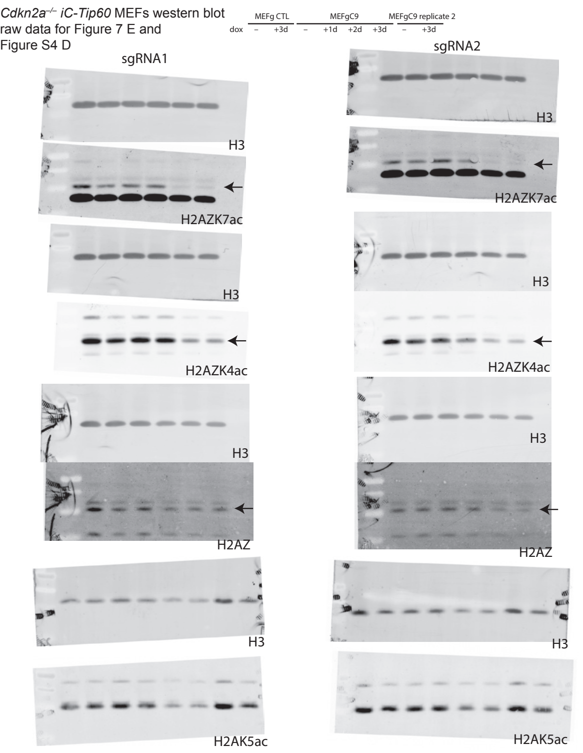

Cdkn2a$^{-/-}$ *iC-Tip60* MEFs western blot raw data for Figure 7 E and Figure S4 D

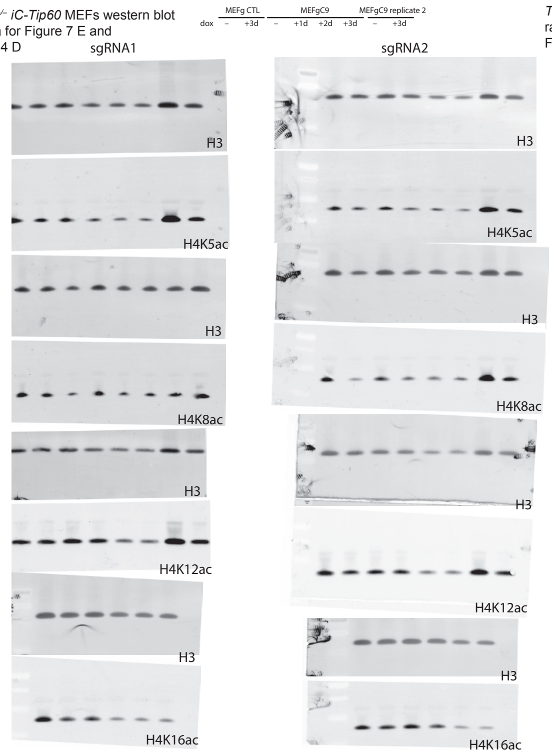

Trp53$^{-/-}$ *iC-Tip60* MEFs western blot raw data for Figure 7 E and Figure S4 D

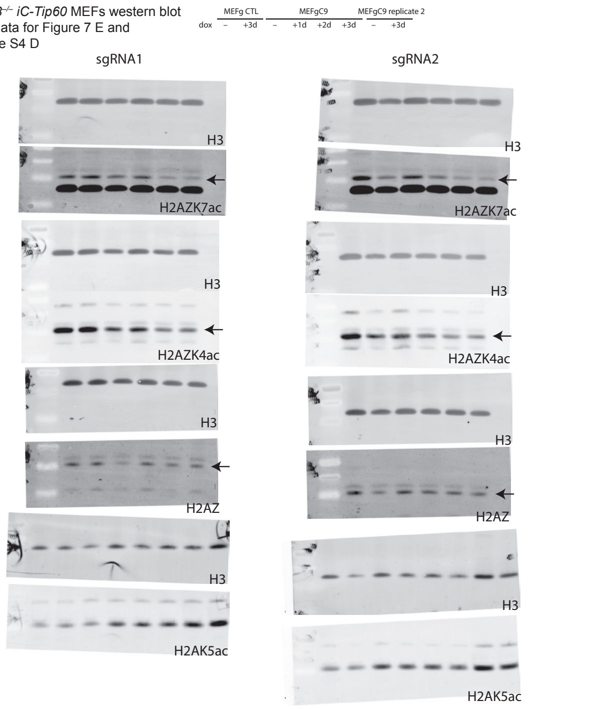

Trp53$^{-/-}$ *iC-Tip60* MEFs western blot raw data for Figure 7 E and Figure S4 D

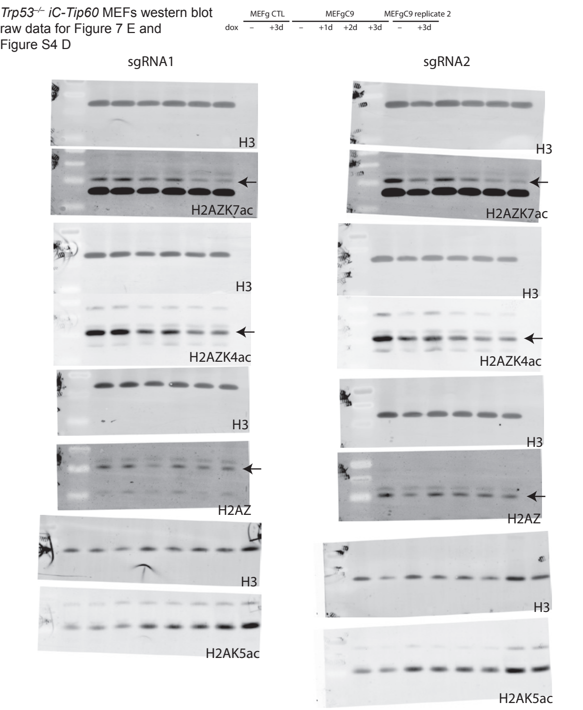

Trp53$^{-/-}$ *iC-Tip60* MEFs western blot raw data for Figure 7 E and Figure S4 D

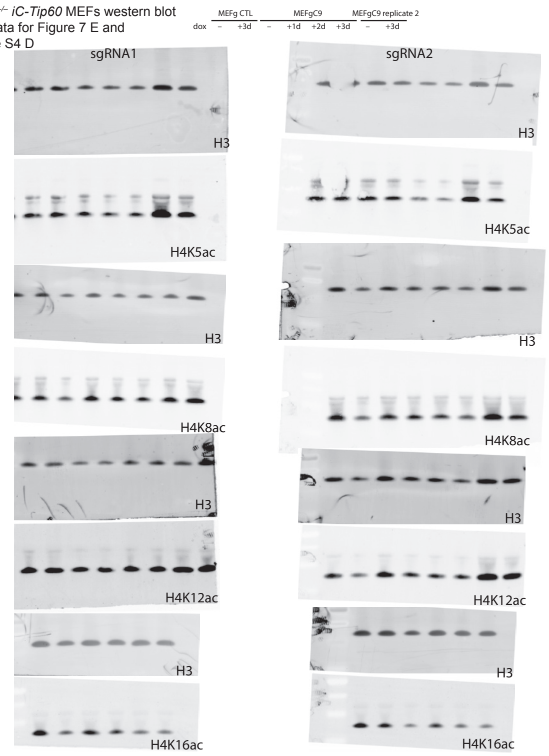

iC-Tip60 MEF

iC-Tip60 MEFs western blot raw data for Figure 3 D and Figure S4 B

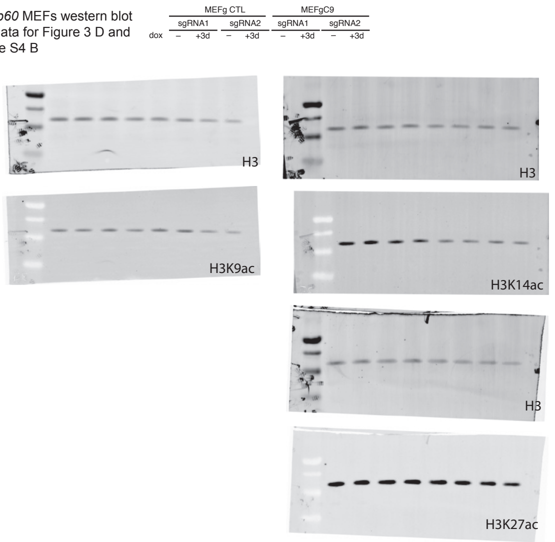

iC-Tip60 MEFs western blot raw data for Figure 3 D and Figure S4 B

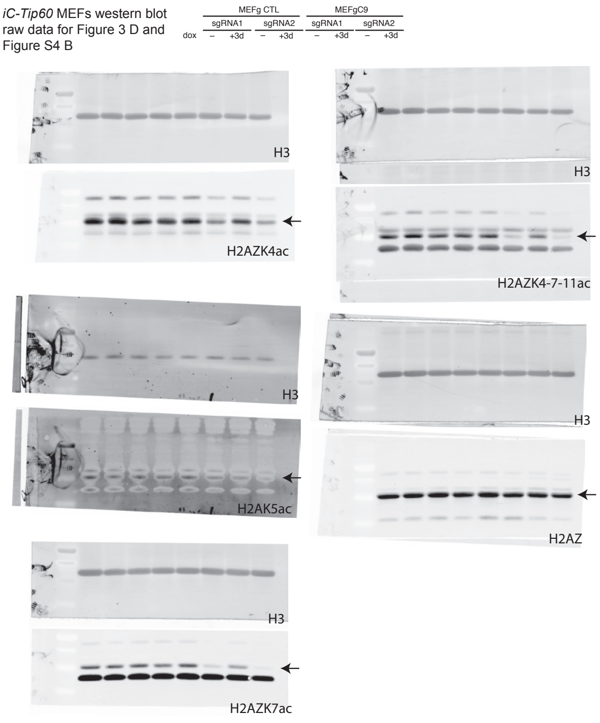

iC-Tip60 MEFs western blot raw data for Figure 3 D and Figure S4 B

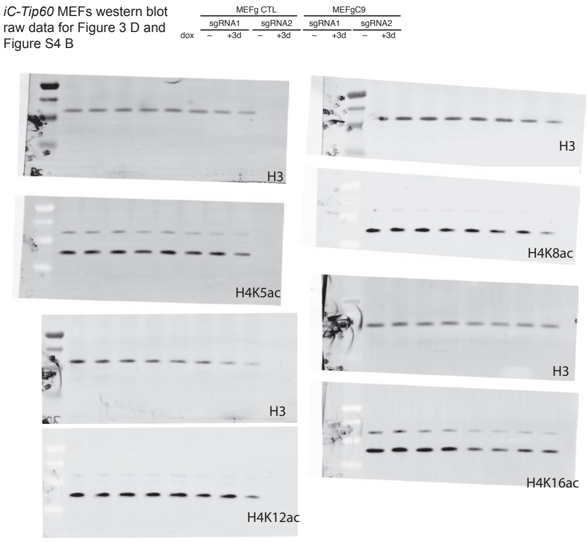

iC-Tip60 MEFs western blot raw data for Figure 3 D and Figure S4 B

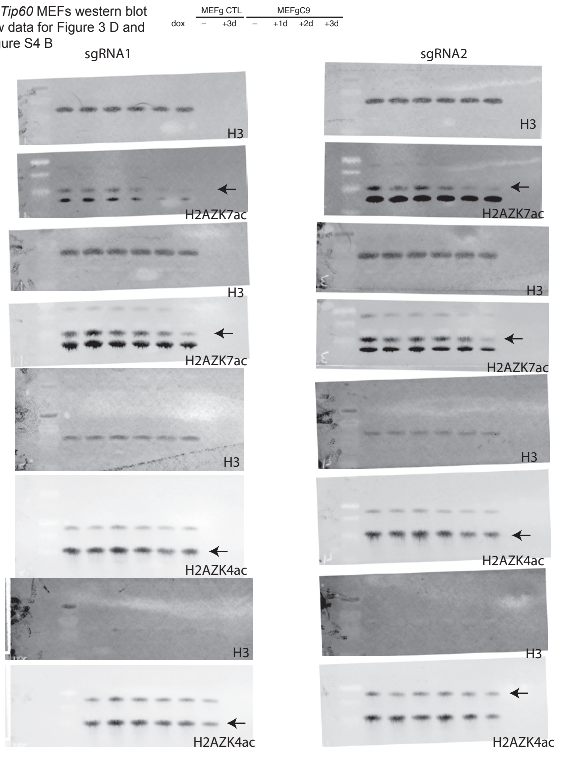

iC-Tip60 MEFs western blot raw data for Figure 3 D and Figure S4 B

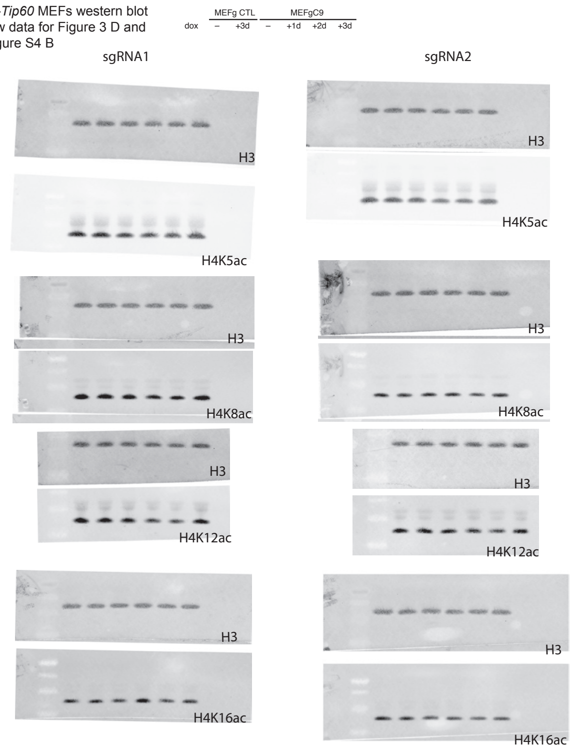

iC-Tip60 MEFs western blot raw data for Figure 3 D and Figure S4 B

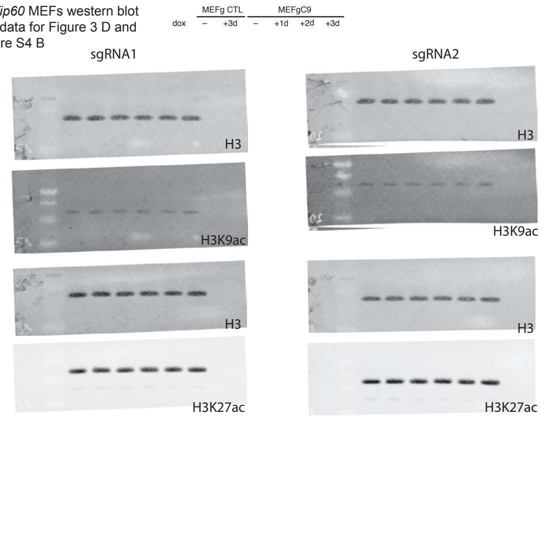

iC-Tip60 MEFs western blot raw data for Figure 3 D and Figure S4 B

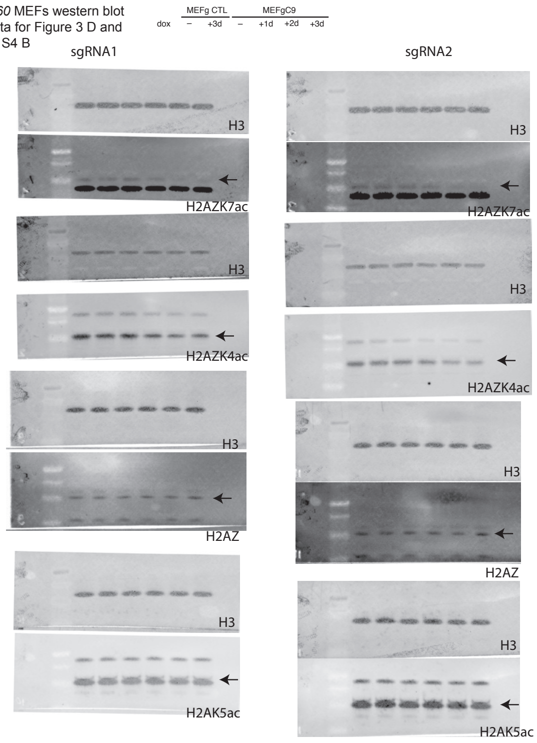

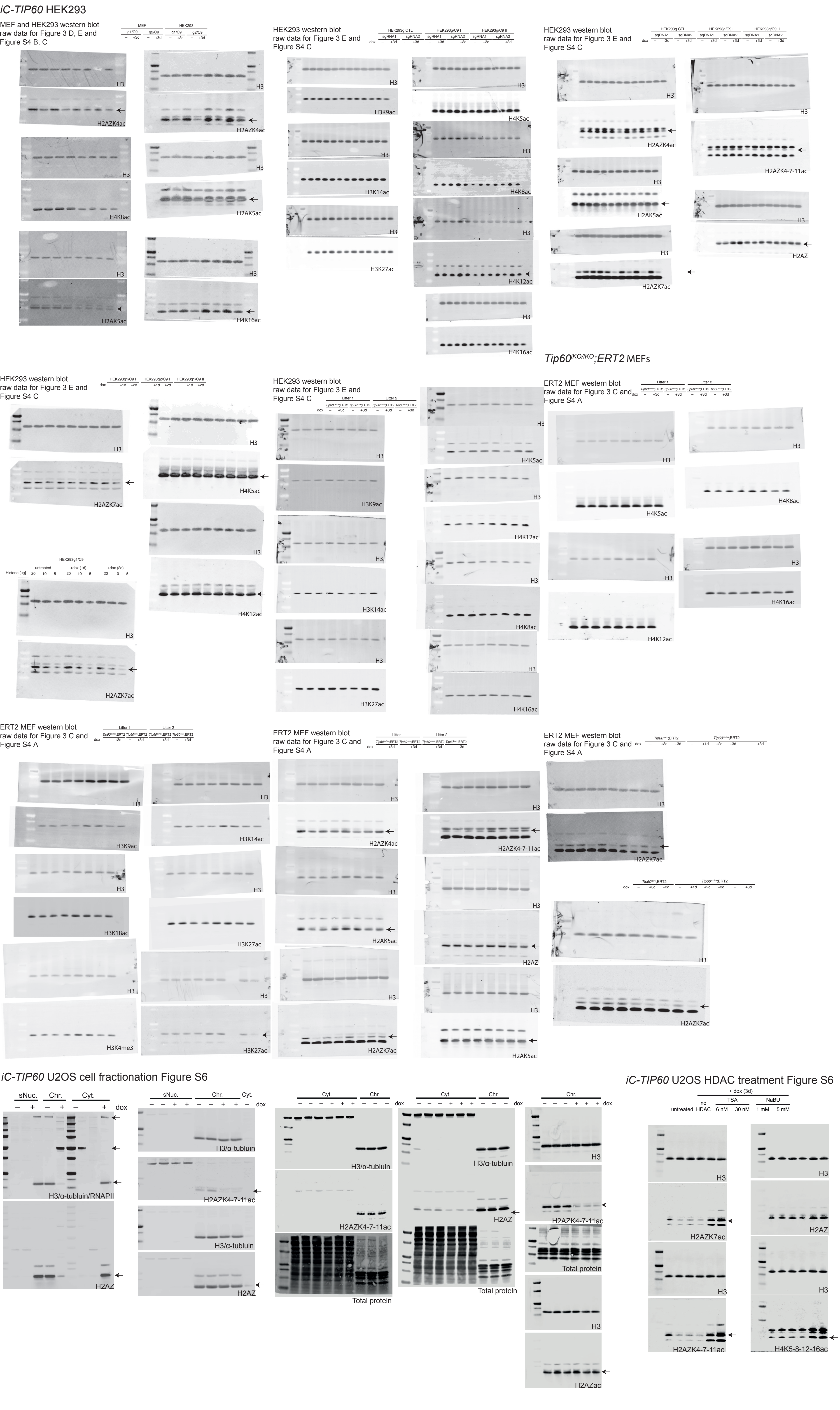

iC-TIP60 HEK293
MEF and HEK293 western blot raw data for Figure 3 D, E and Figure S4 B, C
MEF
HEK293
g1/C9
g2/C9
H3
H2AZK4ac
H4K8ac
H2AK5ac
H4K16ac
HEK293 western blot raw data for Figure 3 E and Figure S4 C
HEK293g CTL
HEK293g/C9 I
HEK293g/C9 II
sgRNA1
sgRNA2
dox
H3K9ac
H4K5ac
H3K14ac
H4K8ac
H3K27ac
H4K12ac
H2AZK4-7-11ac
H2AZ
H2AZK7ac
Tip60iKO/iKO;ERT2 MEFs
HEK293g1/C9 I
HEK293g2/C9 I
HEK293g1/C9 II
Histone [ug]
untreated
+dox (1d)
+dox (2d)
Litter 1
Litter 2
ERT2 MEF western blot raw data for Figure 3 C and Figure S4 A
H3K9ac
H3K14ac
H3K18ac
H3K27ac
H3K4me3
iC-TIP60 U2OS cell fractionation Figure S6
sNuc.
Chr.
Cyt.
dox
H3/α-tubluin/RNAPII
H3/α-tubluin
Total protein
H2AZac
iC-TIP60 U2OS HDAC treatment Figure S6
+ dox (3d)
untreated
no HDAC
TSA
6 nM
30 nM
NaBU
1 mM
5 mM
H4K5-8-12-16ac
